# On the Ordering Mechanism of Cu^+^ in 2D van der Waals Multiferroic CuCrP_2_S_6_

**DOI:** 10.1002/advs.202524227

**Published:** 2026-02-04

**Authors:** Jiasen Guo, Yongqiang Cheng, Michael A. Susner, Ryan P. Siebenaller, Zachary Morgan, Feng Ye

**Affiliations:** ^1^ Neutron Scattering Division Oak Ridge National Laboratory Oak Ridge USA; ^2^ Materials and Manufacturing Directorate, Air Force Research Laboratory Wright‐Patterson Air Force Base Ohio USA; ^3^ Department of Materials Science and Engineering The Ohio State University Columbus USA

**Keywords:** ferroelectrics, incommensurate modulation, neutron and X‐ray scattering, order‐disorder transition, pair distribution function, van der Waals material

## Abstract

CuCrP_2_S_6_ is a van der Waals multiferroic where the tunable Cu^+^ sublattice underpins its exceptional ferroelectric and electronic switching properties. Yet, the microscopic mechanism governing Cu^+^ ordering has remained elusive. Here, we combine single‐crystal X‐ray and neutron diffraction with pair distribution function analysis to uncover a temperature‐driven evolution of Cu^+^ ordering, giving rise to an incommensurate quasi‐antipolar phase between the paraelectric and antiferroelectric states. The modulation originates from correlated Cu^+^ occupancy redistribution coupled to *breathing* distortion of surrounding S_3_ triangles, establishing a symmetry‐adapted lattice distortion mode. Diffuse scattering persisting over 35 K above the transition confirms that the structural instability follows an order‐disorder mechanism. The spontaneous off‐centering of Cu^+^ positions CuCrP_2_S_6_ as a model platform for correlated order‐disorder phenomena in 2D layered ferroics, and provides design principles for next‐generation memory and logic devices.

## Graphical abstract

Temperature‐dependent X‐ray single‐crystal diffuse scattering measurements of CuCrP_2_S_6_ in the (H, K, 0) plane reveal the gradual emergence and strengthening of short‐range order upon cooling from 230 to 170 K. At intermediate temperatures, diffuse features become pronounced and eventually lock into sharp incommensurate satellite reflections, indicating the development of long‐range incommensurate order.

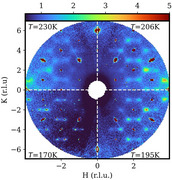

## 1 Introduction

Two‐dimensional (2D) materials open unprecedented opportunities for device miniaturization and energy efficiency in next‐generation electronics. Among them, the quaternary 2D van der Waals (vdW) metal thio/selenophosphate (MTP/MSP) M^I^M^III^P_2_X_6_ (M^I^ = Ag^+^, Cu^+^, M^III^ = Cr^3+^, V^3+^, In^3+^, Bi^3+^ etc., X = S, Se) have risen as a versatile platform for device engineering [1, 2, 3]. Their lamellar layers, built from MX_6_ and P_2_X_6_ octahedra arranged in triangular lattices or parallel strips depending on the M^I^/M^III^ size ratio [4] enable rich structural tunability. The compositional flexibility of these materials yields a diverse range of physical properties spanning optoelectronic responses [5], anisotropic electrical conductance [6, 7], and ferroelectric switching [8, 9], making them ideal candidates for multifunctional nanoelectronic devices. In particular, CuCrP_2_S_6_ (CCPS) stands out as a star compound with its coexisting antiferroelectricity and antimagnetism, along with the strong polarization magnetization coupling [6, 10, 11, 12, 13, 14].

**FIGURE 1:**
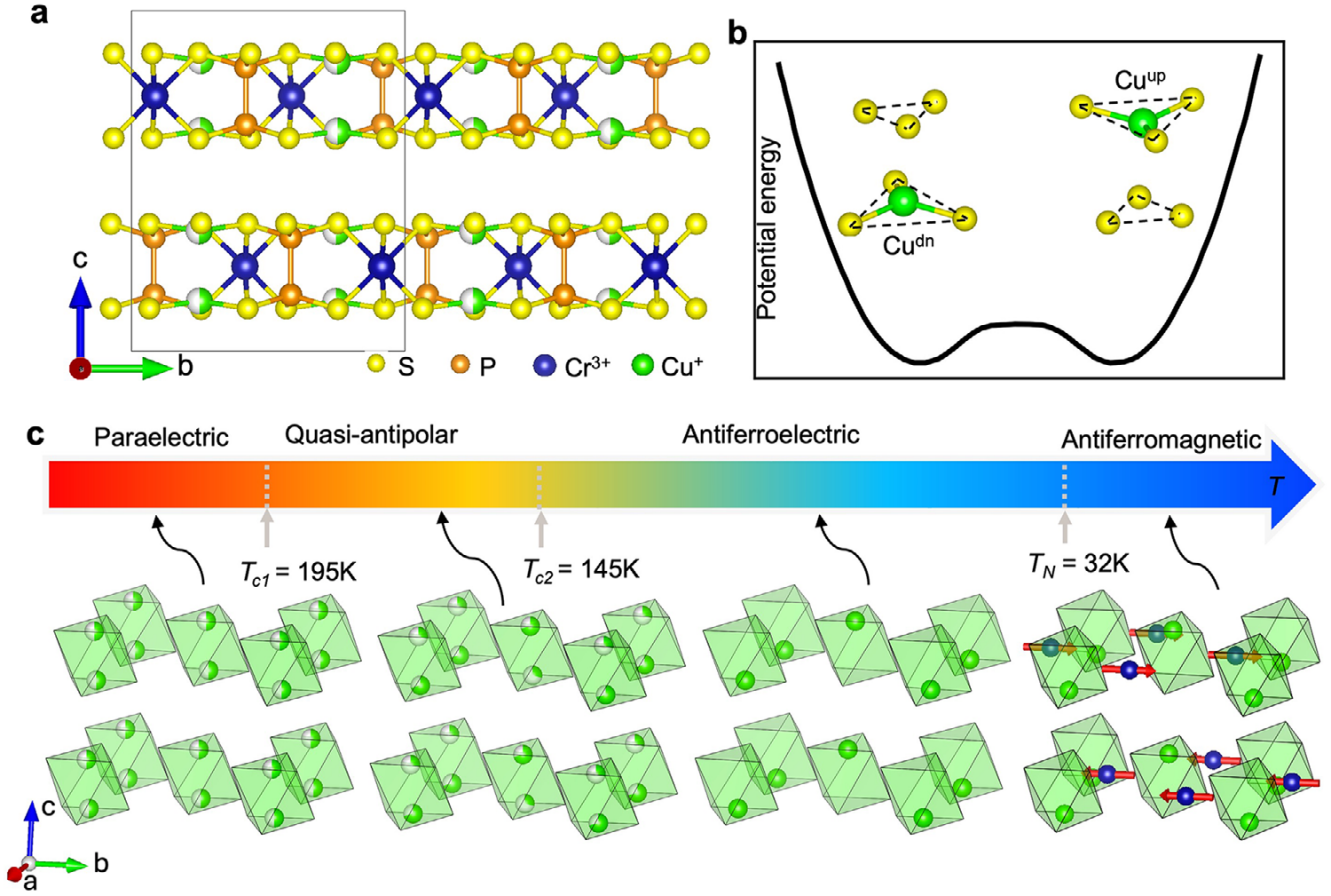
CuCrP_2_S_6_ crystal structure and its temperature evolution of structural (Cu^+^) and magnetic phases (Cr^3+^). (a) Side view of the crystal structure at room temperature, demonstrating lamellar layers stacking along the c‐axis. (b) A schematic illustration of the up and down Cu^+^ positions in a double‐well potential within a single CuS_6_ octahedron. (c) Structural phases and magnetic phases as a function of temperature. For clarity, only the Cu^+^ and Cr^3+^ are shown. Partially‐filled spheres are used to show Cu^+^ occupation, and red arrows denote Cr^3+^ magnetic moments.

At ambient conditions, CCPS crystallizes in a monoclinic structure (space group C2/c) [15] composed of stacked layers along the c axis, separated by vdW gaps (Figure 1a). Each layer contains edge‐sharing CrS_6_, P_2_S_6_ and CuS_6_ octahedra that form interconnected triangular networks. The coexistence of Cu^+^ and Cr^3+^ ions is crucial for realizing both ferroelectric and magnetic orders. The closed‐shell Cu^+^ ($d^{10}$) exhibits a strong tendency toward off‐center displacement driven by the pseudo‐Jahn‐Teller effect (PJTE) [16, 17, 18, 19], enhancing the hybridization of Cu^+^ 4s and 3d orbitals within trigonal coordination. Diffraction measurements inferred that Cu^+^ cations reside in an effective double‐well potential, favoring sites near the up and down S_3_ triangles [15, 18, 20]. This bistable configuration gives rise to highly tunable Cu^+^ sublattice that can be reversibly polarized under an electric field, making CCPS a promising ferroelectric medium with switchable dipole states [8, 9, 21, 22].

The trivalent Cr^3+^ ($d^{3}$) ion, on the other hand, is centered within its octahedron, distinct from the off‐centering In^3+^ ($d^{10}$) in the isostructural CuInP_2_S_6_ (CIPS) [18]. The localized magnetic moment of Cr^3+^ (3.8$\mu_{B}$) introduces an additional spin degree of freedom, coupling magnetism to the polar Cu^+^ sublattice. Because the ferroelectric polarization originates from Cu^+^ displacements while the magnetism arises from Cr^3+^ spins, CCPS is classified as a type‐I multiferroic [23]. Remarkably, it also exhibits magnetoelectric coupling, typically associated with type‐II multiferroics [11, 13], endowing CCPS with dual ferroic controllability.

Extensive investigations have been devoted to elucidating how the Cu^+^ sublattice in CCPS governs its ferroic behavior [6, 8, 9, 24, 25]. Recent studies have demonstrated electric‐field tunable ferroelectricity persisting above room temperature [8, 9], yielding enhanced switching endurance and thermal stability compared to CIPS (120 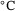 vs. 42 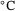) in developing non‐volatile memory devices [8]. Tunable local ferroelectricity below T = 145 K has been attributed to the defect‐induced dipoles within the vdW gap [25], while in‐plane anisotropic conductance was linked to Cu^+^ migration within the individual lamellar layer — an effect favorable for artificial synapses and neuromorphic devices [6]. Moreover, unconventional magnetoelectric coupling driven by Cu^+^‐induced lattice distortion has been proposed [14], further underscoring the intricate interplay among charge, lattice, and spin. These discoveries highlight the critical role of the Cu^+^ sublattice in dictating the wide spectrum of electronic and magnetic functionalities in CCPS.

At high temperatures (T>195 K), CCPS adopts a paraelectric (PE) state (Figure 1c), in which the Cu^+^ ions dynamically rattle between the upper and down sites (${\mathrm{Cu}}^{\mathrm{up}}$ and ${\mathrm{Cu}}^{\mathrm{dn}}$) within their ${\mathrm{CuS}}_{6}$ octahedra, resulting in a split occupancy with no net polarization. Upon cooling below approximately 145 K, long‐range antiferroelectric (AFE) order develops, characterized by alternating local polarizations between adjacent CuS_6_ octahedra connected by a [1/2,1/2,0] translation vector within each lamellar layer that repeats across the vdW gaps. Between these two regimes lies an intermediate quasi‐antipolar state, distinguished by partial Cu^+^ ordering and is often described as a “glassy precursor” to the AFE state. At still lower temperature $T_{N}$ = 32 K, the Cr^3+^ spins order antiferromagnetically (AFM) in an A‐type configuration, with ferromagnetically aligned moments confined in the ab‐plane.

Even with substantial experimental advances, a clear picture of the quasi‐antipolar state in CCPS has not emerged. Earlier neutron powder diffraction at 160 K revealed an approximately 2:1 occupancy ratio between ${\mathrm{Cu}}^{\mathrm{up}}$ and ${\mathrm{Cu}}^{\mathrm{dn}}$ sites [26], while calorimetric studies suggested the presence of an incommensurate (ICM) phase, analogous to that observed in Sn_2_P_2_Se_6_ [27]. Although it was inferred from bulk measurements [28] and proposed for a related compound CuInP_2_Se_6_ [29], direct structural evidence has so far been lacking. Furthermore, the nature of the transition from the PE state to the quasi‐antipolar state is under debate based on bulk measurements [26, 30]. Elucidating the ordering mechanism of the Cu^+^ sublattice is therefore essential to comprehend the aforementioned functionalities, which could further inform the implications on other fronts including multi‐state memories [31, 32, 33] and adaptive energy storage [34], where precise control of FE–AFE transitions is critical.

In this paper, we combined single crystal X‐ray and neutron diffraction with 3D difference pair distribution function (3D‐ΔPDF) analysis to reveal the temperature‐dependent evolution of Cu^+^ ordering in CCPS. Our results provided the first experimental evidence of an ICM quasi‐antipolar state between the PE and AFE states. It features a synchronized modulation of Cu^+^ occupancy and the surrounding S_3_ matrix, effectively dictated by a symmetry‐constrained structural distortion. Notably, broad diffuse scattering is present well above the ICM transition in the PE state. 3D‐ΔPDF analysis further shows temperature‐independent local structural distortions, clarifying the order‐disorder mechanism for the ICM transition.

## 2 Results and Discussion

**FIGURE 2:**
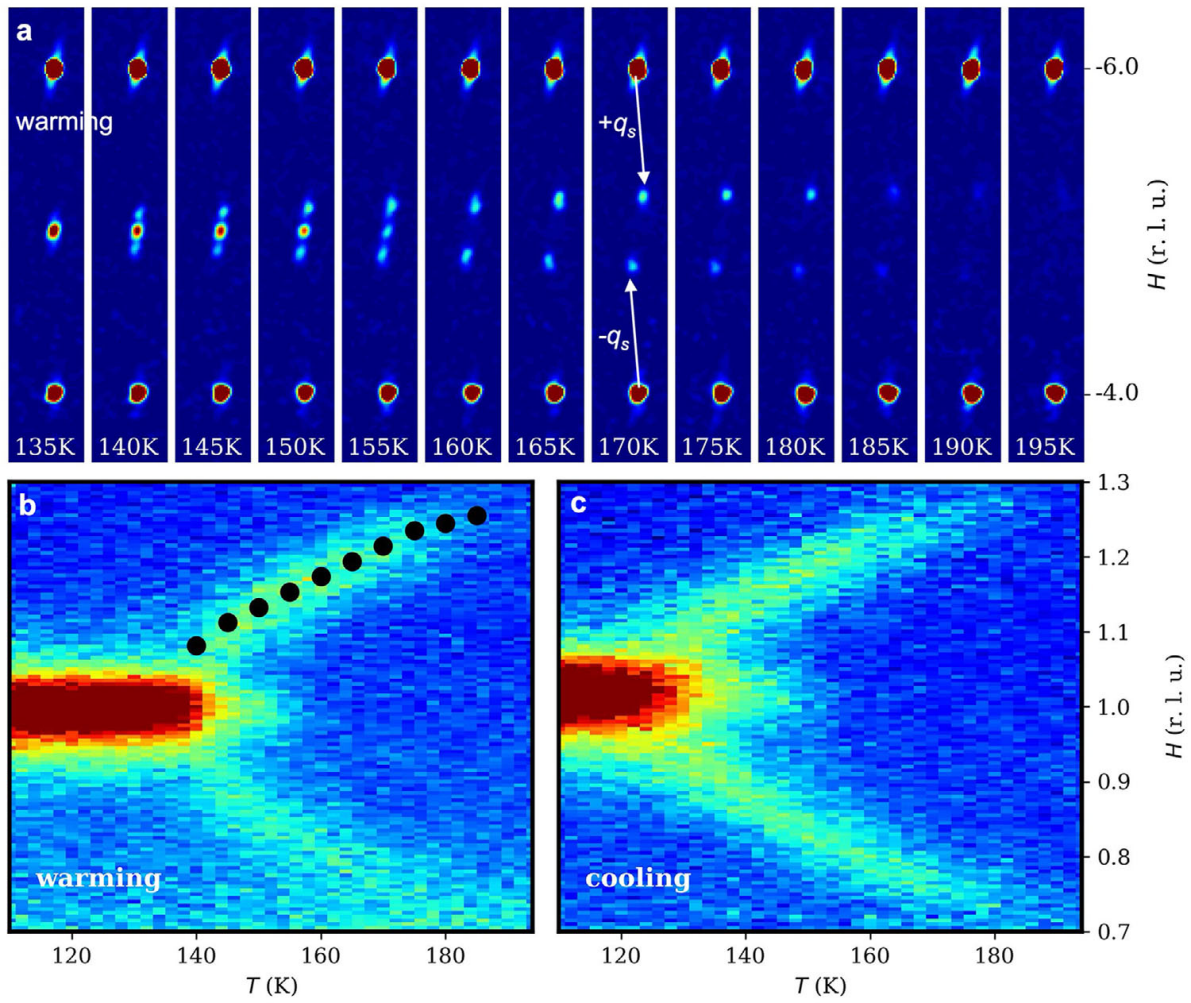
The temperature evolution of the ICM structural modulation revealed by single crystal X‐ray and neutron diffraction measurements. (a) X‐ray scattering patterns in the (H,0,L) plane at selected temperatures on warming. White arrows indicate the modulation wavevector $q_{s}$. The temperature dependence of the scattering near the structural peak (1,0,−2) from neutron diffraction on (b) warming and (c) cooling. Solid black symbols are the $a^{★}$ component of $q_{s}$ from the X‐ray data.

Single‐crystal X‐ray diffraction measurements show a clear sequence of structural transitions in CCPS upon cooling. Figure 2a displays the diffraction pattern in the (H,0,L) plane collected at selected temperatures. At $T_{c1}∼195$ K, weak satellite peaks emerge at $q_{s}=\left(\pm \alpha ,0,\pm \gamma \right)$, signaling the onset of structural modulation as it occurs well above the known magnetic order. The modulation involves primarily the Cu^+^ sublattice, which dominates in X‐ray scattering cross section (Cu > Cr ≫ S ≫ P). The gradual appearance and sharpening of these satellite peaks indicate a continuous, second‐order phase transition, consistent with thermal dynamic observations [26]. The absence of reflections with H+K=2n+1 implies the retention of C‐centering, which symmetrizes CuS_6_ octahedra within each lamellar layer.

Upon further cooling, the satellite peaks shift in position and intensify. Below $T^{★}$ = 155 K, additional reflections appear at commensurate (CM) positions. The lock‐in transition is first‐order where the ICM quasi‐antipolar phase coexists with the CM AFE state, align with the specific‐heat and dielectric studies [25, 26]. The new reflections appear at H+K=2n+1, L=2m, which are forbidden in the PE phase (S.G. C2/c). As temperature decreases further, intensity transfers from the satellites to the CM reflections, indicating a change in the relative volume fractions. The satellites vanish completely at $T_{c2}$ = 135 K, marking the completion of the transition. Neutron diffraction measurements further corroborate the structural evolution (Figure 2b,c). The diffraction intensity of the (1,0,−2) peak exhibits a pronounced thermal hysteresis between cooling and warming, typical for first‐order transition.

**FIGURE 3:**
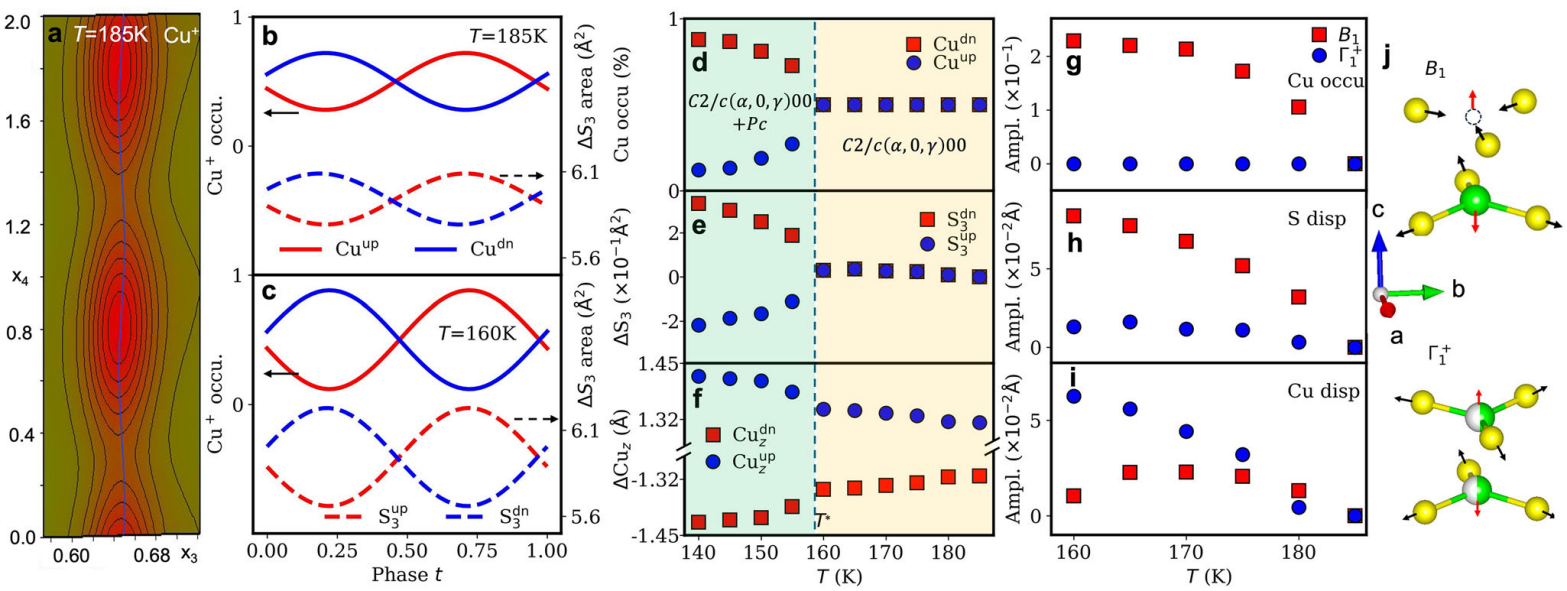
The structural modulation in the ICM quasi‐antipolar state and corresponding symmetry distortion modes. (a) $x_{3}$‐$x_{4}$ section of the Cu^+^ superspace electron density at T= 185 K. Red and green color represent positive and negative electron density, and solid black lines are the isocontours. The solid blue line is the refined atomic modulation function. Refined modulation functions of the Cu^+^ site occupancies (solid lines), and the nearby triangular area $S_{3}^{\mathrm{up}}$ and $S_{3}^{\mathrm{dn}}$ (dashed lines) as functions of modulation phase t (b) 185K and (c) 160 K. Thermal evolution of (d) the site occupancies of ${\mathrm{Cu}}^{\mathrm{up}}$/${\mathrm{Cu}}^{\mathrm{dn}}$, (e) the relative change of $S_{3}^{\mathrm{up}}$/$S_{3}^{\mathrm{dn}}$ area, and (f) the z coordinate of ${\mathrm{Cu}}^{\mathrm{up}}$/${\mathrm{Cu}}^{\mathrm{dn}}$ relative to the center position. All quantities refer to the average structure. The definition of up and down is relative. Temperature dependence of decomposed local structural distortion amplitudes of (g) Cu^+^ occupancy, (h) S displacement, (i) Cu^+^ displacement. The amplitudes are plotted as the relative changes from the high temperature values. (j) Schematic illustrations of the two distortion modes for Cu^+^ and S. Black and red arrows denote displacements for S and Cu^+^. Open, half‐ and filled circles refer to empty, partial and occupied Cu^+^ site, respectively.

To elucidate the ICM modulation, we performed a (3+1)‐dimensional superspace refinement using jana2020 [35]. The analysis identifies the ICM phase as a structure with superspace group C2/c(α,0,γ)00, featuring both occupational modulation of the Cu^+^ sublattice and correlated displacive modulation of the surrounding $S_{6}$ matrix. Figure 3a displays the refined superspace electron density section in the $x_{3}$‐$x_{4}$ plane for the ${\mathrm{Cu}}^{\mathrm{up}}$ site at T= 185 K, where $x_{1}$, $x_{2}$, $x_{3}$ are the internal coordinates in the superspace, and $x_{4}$ is determined through the modulation phase t (Additional details are explained in the Supporting Information). The alternating hills and valleys along $x_{4}$ reflect periodic variation in Cu^+^ occupancy. These variations are well described by complimentary harmonic functions, maintaining an overall unity Cu^+^ occupancy within each CuS_6_ octahedron (Figure 3b). Concurrently, the atomic modulation of S generates *breathing* distortions of the S_3_ triangle (Figure 3b and Figure S1). This S_3_ displacement, along with Cu^+^ occupancy, reflects a local lattice expansion to accommodate off‐centered Cu^+^ ions and to minimize the total energy. The amplitude of the ICM modulation continues to grow with decreasing temperature, while the average value—integration over one modulation period—is essentially constant (Figure 3c). Notably, the preferential occupation of the ${\mathrm{Cu}}^{\mathrm{up}}$ or ${\mathrm{Cu}}^{\mathrm{dn}}$ sites is never realized in the ICM phase. The two Cu^+^ sites and their surrounding S_3_ triangles stay statistically equivalent, preserving the averaged C2/c symmetry. Below $T_{c2}$, the system is refined in a polar space group Pc, in agreement with the neutron powder diffraction and the second‐harmonic generation (SHG) measurements [13, 15].

The temperature dependence of the average Cu^+^ site occupancies and the S_3_ triangular area (Figure 3d,e) follow a two‐stage behavior with a clear bifurcation at $T^{★}$. Above $T^{★}$, the overall C2/c symmetry requires equal ${\mathrm{Cu}}^{\mathrm{up}}$/${\mathrm{Cu}}^{\mathrm{dn}}$ occupancy and constant S_3_ area, with weak negative thermal expansion (NTE) (Figure S2). Below $T^{★}$, a CM phase emerges and coexists with the ICM one, where one of the two Cu^+^ sites is fully occupied. Overall, this results in an imbalance of the average Cu^+^ site occupation between the two sites, accompanied by expansion/contraction of the surrounding S_3_ triangles. In comparison, Figure 3f illustrates a smoother shift of Cu^+^ along the layer normal across both temperature ranges, exhibiting minimal sensitivity to $T^{★}$. This difference between Cu^+^ occupancy and displacement indicate the involvement of multiple distortion modes.

Detailed distortion subgroup analysis is conducted using ISODISTORT [36, 37]. Each local structural distortion, Cu^+^ occupancy/displacement variation and S displacement, can be decomposed into two symmetry‐adapted modes: $B_{1}\left(\alpha ,0,\gamma \right)$ and $\Gamma_{1}^{+}\left(0,0,0\right)$. The $B_{1}$ mode describes local symmetry breaking with Cu^+^ redistribution between the up and down sites and anti‐phase *breathing* of the surrounding S_3_ triangles, along with an anti‐phase Cu^+^ shift with respect to neighboring CuS_6_ perpendicular to the layer. The $\Gamma_{1}^{+}$ mode describes an overall layer expansion/contraction (strain) with in‐phase Cu^+^ shift between neighboring CuS_6_ (Figure 3j). With decreasing temperature, the $B_{1}$ mode shows a substantial enhancement in Cu^+^ occupancy variation and S displacement, while only negligible participation of the $\Gamma_{1}^{+}$ mode in S displacement is observed due to layer expansion (Figure 3g,h). Conversely, Cu^+^ displacement involves both modes and the $\Gamma_{1}^{+}$ mode becomes the dominant one as temperature reduces (Figure 3i). Thus, Cu^+^ redistribution and S_3_ anti‐phase *breathing* are driven by local C2/c symmetry breaking. It is worth noting that the Cu^+^ displacement (∼ 1% along layer normal) could be the driving mechanism for the NTE effect (∼ 0.1% along c); the closer Cu^+^ across the vdW gap leads to an increased overall electrostatic repulsion between the lamellar layers and expands the lattice spacing.

The refinement between the PE and AFE states confirms the presence of a modulated structure. Such successive ICM‐CM transitions are well known in classical ferroelectrics, including NaNO_2_, ${\left({\mathrm{NH}}_{4}\right)}_{2}{\mathrm{BiF}}_{4}$, and K_2_SeO_4_ [38, 39, 40, 41]. In general, structural transitions can be classified as either order‐disorder or displacive types [42]. In the former case, the transition is governed by relaxational dynamics, where local structural distortions reside in multi‐well potentials that become increasingly correlated upon approaching the transition [42, 43]. The apparent high symmetry of the disordered phase arises from thermally assisted hopping between potential wells. In contrast, a displacive transition arises from small, continuous atomic displacements from equilibrium positions, characterized by the condensation of a soft phonon mode at a superlattice wavevector [27, 29, 39, 44, 45, 46, 47, 48, 49]. The disordered phase retains high symmetry associated with a single potential well that evolves with temperature. In a pure displacive transition, the soft phonon frequency is expected to approach zero at the transition [38], whereas in an order‐disorder transition, the frequency remains finite [50].

This distinction has been explored through Raman spectroscopy studies in CCPS, which suggests an order‐disorder mechanism [25]. The Cu^+^‐related low‐frequency Raman mode follows a mean‐field relation $\omega^{2}\propto \left(T-T_{c1}\right)+\omega_{0}^{2}$, maintaining a finite frequency $\omega_{0}$ at $T_{c1}$ [25], which was also observed in a separate Raman work [51]. This mode likely corresponds to the amplitudon of the modulated structure—that is, the vibration of its amplitude—with an eigenvector described by the $B_{1}$ distortion. As the temperature decreases, it gradually hardens and becomes a Γ‐point mode in the AFE state. Density functional theory calculations predict that this Γ‐point mode at ∼ 48 ${\mathrm{cm}}^{-1}$ involves a S_3_ *breathing* motion coupled with the Cu^+^ displacement along the layer normal, in line with the $B_{1}$ distortion illustrated in Figure 3j (Figure S9).

The above assignment of the order‐disorder mechanism generally applies to CM transitions, where structural instabilities occur at q=0. For an intrinsic displacive ICM transition with wavevector $q_{s}$, a finite phonon gap would appear at q=0 in Raman spectroscopy, which could lead to a transition being mistakenly identified as order‐disorder type [52]. However, a nonzero gap has also been observed in the displacive compound Sn_2_P_2_Se_6_ even at $q_{s}$ [53]. Such incomplete phonon softening has been attributed to an intrinsic crossover from displacive to order‐disorder behavior as the transition is approached [42]. It is now widely accepted that most materials exhibit characteristics of both mechanisms.

The presence of local structural distortions in the disordered phase is the key to distinguish the two. Although deviations of Cu^+^ distribution from the average structure were inferred from X‐ray [20] and neutron [15] diffraction, experimental evidence probing locally disordered Cu^+^ positions in the PE state have not yet been reported. X‐ray diffuse scattering and pair‐distribution function analysis [54, 55, 56] have proven powerful for detecting local structural correlations, providing a complementary approach to X‐ray absorption fine structure [43] and nuclear magnetic resonance measurements [57].

**FIGURE 4:**
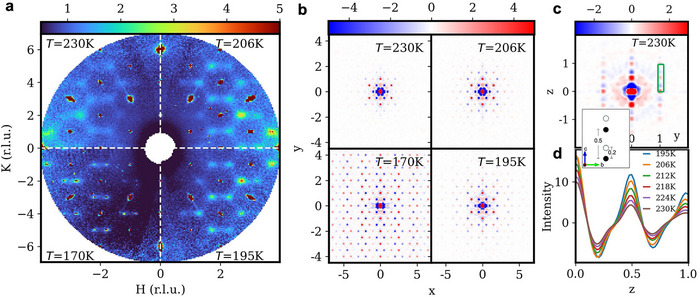
X‐ray diffuse scattering and 3D‐ΔPDF analysis. (a) Diffuse scattering in the (H,K,0) plane at 230 K, 206 K, 195 K and 170 K, respectively, arranged clockwise. (b) The corresponding 3D‐ΔPDF patterns in the ab‐plane in fractional coordinates of the real‐space unit cell. Positive and negative correlations are colored in red and blue. (c) 3D‐ΔPDF patterns in the bc‐plane in fractional coordinates at 230 K, and (d) the peak intensity profiles along the c‐axis at sequential temperatures in the marked region of interest. Due to the monoclinic angle, the intensity at −0.02<x<0.14 are integrated to include the inter‐ and intra‐CuS_6_ correlations in the same plot. The inset shows the Cu^+^ site arrangement within the unit cell along the layer normal with z separations labeled. Filled and open circles illustrate occupied and empty sites in the AFE state. Intensities in all plots are in arbitrary units.

Figure 4a shows X‐ray scattering patterns in the (H,K,0) plane collected at 230, 206, 195, and 170 K. Pronounced diffuse scattering is readily visible at T = 230 K, indicating significant short‐range correlations in the ${\mathrm{Cu}}^{+}$ sublattice, roughly 35 K above $T_{c1}$. As the temperature decreases toward $T_{c1}$, diffuse intensities condense into satellite reflections of the ordered phase. Quantitative analysis yields a correlation length of ξ∼ 4.6(3) Å along the b‐axis at 230 K which increases rapidly near the transition, reflecting the progressive buildup of the Cu^+^ distribution coherence.

The corresponding 3D‐ΔPDF map further clarifies these correlations in real space (Figure 4b). 3D‐ΔPDF for X‐ray scattering measures the autocorrelation of the difference between the total electron density and the long‐range ordered electron density ($\delta \rho =\rho_{\mathrm{total}}-\rho_{\mathrm{periodic}}$) [58], representing the probability of finding pairs of fluctuating atoms separated by vector r. It is determined through an inverse Fourier transform of the diffuse scattering intensity, given by

(1) $$3D-\Delta \mathrm{PDF}=\langle \delta \rho (r)⊗\delta \rho (r)\rangle =F^{-1}\left[I_{\mathrm{diffuse}}\right]$$

where <> and ⊕ stand for time average and the autocorrelation operator, respectively.Above $T_{c1}$, positive and negative correlation appear between the central Cu^+^ site and its the nearest and the next‐nearest neighbor shells, signifying locally anti‐aligned dipole arrangements. At 230 K, the 3D‐ΔPDF signal decays rapidly within the ab‐plane. A lamellar‐layer correlation length of ξ ∼4.3(2) Å is obtained, which is in an excellent agreement with the reciprocal space analysis. This correlation pattern persists on cooling, with ξ increasing sharply across $T_{c1}$. The PDF map also captures the real space atomic modulation in the ICM quasi‐antipolar state, corroborating with the superspace refinement discussed above.

The 3D‐ΔPDF pattern projected in the bc‐plane at 230 K shows peaks with alternating signs along c‐axis (Figure 4c). Positive peaks at integer and half‐integer correspond to the interlayer separation across the vdW gaps, whereas negative ones are shifted by ∼ 0.2 unit cell — the exact ${\mathrm{Cu}}^{\mathrm{dn}}$‐${\mathrm{Cu}}^{\mathrm{up}}$ separation within the octahedra. These characters support the double‐well potential landscape for Cu^+^ even in the PE phases. The peak amplitudes increase progressively with decreasing temperature.

The combined reciprocal‐ and real‐space analysis establish that the ICM transition in CCPS follows an order‐disorder mechanism, accompanied by persistent local structural distortions. The short‐range Cu^+^ correlations in the PE state and modulated structure in the ICM quasi‐antipolar state highlight a frustrated lattice landscape, where competing interactions prevent a simple transition to an ordered phase. Such a scenario is frequently encountered in ferroelectric systems [59, 60] and analogous to the ICM spin states [61]. Ferroelectric transitions of order‐disorder type are often described using pseudo‐spin lattice models like the axial Ising framework [62, 63]. It captures the interplay between Coulombic repulsion and elastic deformation, stabilizing the modulated phase. Extending such theoretical approaches to the broader family of Cu^+^‐based MTP/MSP [21, 22] could provide critical insights to predict and design functional switching behaviors in 2D ferroic systems.

The tendency of a quasi‐trigonal coordination of Cu^+^ and other closed‐shell ($d^{10}$) cations was widely reported [18, 64, 65] and can be attributed to PJTE within the vibronic coupling framework [16, 17, 66]. In this model, the energy gain arising from lattice distortion Q is expressed as $\Delta E\propto 1/2\left(K_{s}+K_{v}\right)Q^{2}$. Here, $K_{s}$ is the static restoring elastic energy, and $K_{v}$ accounts for charge relaxation due to the displacement‐induced vibronic coupling between the ground state $\Psi_{0}$ and excited state $\Psi_{i}$. $K_{v}$ is inversely proportional to the energy separation, $-1/(E_{i}-E_{0})$. In general, a symmetry‐lowering cation displacement is energetically unfavorable due to restoring forces. However, such displacement could promote hybridization between the cation's d and s orbitals, thereby stabilizing the local distortion. This effect becomes significant for closed‐shell cations such as Cu^+^, Ag^+^, where the small inter‐configurational energy gap ($E_{i}-E_{0}$) between $d^{9}s^{1}$ and $d^{10}$ results in a substantial energy gain [17].

Electronic instabilities have also been identified in open‐shell ($d^{n}$) transition‐metal cations with specific spin states [66]. For instance, low spin (LS) Mn^3+^ ($d^{4}$) in octahedral ligand field is PJTE active owing to an allowed one‐electron transition between the ground and excited states that conserves the spin multiplicity. In contrast, for high spin (HS) Mn^3+^, this excitation is forbidden because it violates the spin‐multiplicity conservation rule, rendering the ion PJTE inactive [66]. Such correlated magnetic and ferroelectric phenomena can be effectively tuned through a spin‐crossover (SCO) process, often triggered by external stimuli such as temperature, pressure and light [67]. For example, the ${\mathrm{Fe}}^{2+}$‐containing SCO compounds have demonstrated reversible transitions between the high temperature HS and low temperature LS states. Similar switches can also be induced via photoexcitation mediated through intermediate states enabled by spin‐orbital‐coupling [67, 68]. Because the LS state exhibits a shorter metal‐ligand bond than the HS one, pressure serves as another knob controlling cationic spin configuration. Therefore, for compounds containing magnetic PJTE active cations, in situ studies combining real‐time perturbations with advanced characterization, such as diffraction and piezoresponse force microscopy will be insightful.

## 3 Conclusion

Our results deliver microscopic evidence of an ICM state between the PE and AFE phases and resolve the ordering mechanism of Cu^+^ in CCPS. The ICM phase is characterized by a correlated structural modulation, where Cu^+^ occupancy redistribution couples with $S_{3}$‐triangle distortion to form an ICM quasi‐antipolar configuration. Notably, local Cu^+^ off‐center distortions and diffuse scattering persist well above the ICM transition, establishing an order‐disorder mechanism that unifies the tunable ferroelectricity, anisotropic transport, and multiferroicity. These correlated order‐disorder phenomena in 2D layered ferroics bridge the physics of classical perovskite ferroelectrics with emerging van der Waals systems, offering new design strategies for next‐generation low‐dimensional functional devices. The direct manipulation of the Cu^+^ ordering through external stimuli represents one key approach. Moreover, the chemical flexibility of the broader MTP/MSP family allows a similar polar order to be realized by substituting other PJTE active cations such as Ag^+^ or ${\mathrm{Cd}}^{2+}$. Finally, compounds incorporating magnetic PJTE active cations—where polar and magnetic orders coexists at a common site—open promising avenues toward novel multifunctional properties that merit future exploration.

## 4 Experimental Section

### 4.1 Sample Synthesis

Single crystal CCPS were synthesized from pure elements following the method described in Ref. [24].

### 4.2 X‐Ray Diffraction

The X‐ray measurements were carried out on a single crystal of CCPS flake with dimensions of 80×80×5 $\mu m^{3}$ mounted to a Mitegen loop using N‐type vacuum grease. Data were collected on a Rigaku Synergy‐DW system with source Mo wavelength 0.7107 Å with temperature regulated using Oxford cryostream between 100 and 255 K. At each temperature, the reduced data were processed with the CrysAlisPro software.

### 4.3 Neutron Diffraction

Neutron diffraction was performed on the time‐of‐flight single crystal diffractometer CORELLI at the Spallation Neutron Source (SNS) at Oak Ridge National Laboratory (ORNL) [69]. CCPS single crystals of a total mass ∼8 mg were co‐aligned in the ab‐plane on an aluminum plate mounted to a sample stick inserted in a closed‐cycle refrigerator with a base temperature ∼4 K. A large volume of reciprocal space was surveyed by rotating the sample stick 360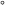 at 3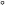 steps at each temperature, with 2‐min data collection per angle. Temperature evolution of the structure was characterized via monitoring characteristic peaks at fixed angles through ramping the temperature with a constant warming rate.

### 4.4 Density Functional Calculation

Spin‐polarized density functional theory (DFT) calculations were performed using the *Vienna Ab initio Simulation Package* (VASP) [70]. The projector augmented‐wave (PAW) method [71, 72] was employed to describe the core–electron interactions, with a plane‐wave energy cutoff of 800 eV for the valence electrons. The lattice parameters, atomic coordinates, and magnetic structure obtained in this work were used as the initial configuration. The unit cell contained 40 atoms, including four Cr atoms. A Hubbard U correction of 3.7 eV was applied to account for the localized 3d electrons of Cr [73]. The electronic structure was calculated on a 6×3×2 Γ‐centered k‐point mesh. The convergence criteria for electronic and ionic relaxations were set to 10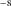 and 10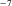 eV, respectively, with the maximum residual interatomic force after relaxation below 0.001 eV/Å. Dispersion interactions were treated using the optB86b‐vdW functional [74, 75]. Density functional perturbation theory (DFPT), as implemented in VASP, was used to compute the Γ‐point phonons and macroscopic dielectric tensor. The Raman activity of each phonon mode was subsequently evaluated using the *vasp_raman* script developed by Fonari and Stauffer [76]. Gaussian broadening was applied to the calculated Raman intensities to generate the simulated Raman spectrum, and the frequencies were scaled by a factor of 1.06 to match the experimental data, accounting for systematic computational errors.

## Conflicts of Interest

The authors declare no conflicts of interest.

## Supporting information


**Supporting File 1**: advs74180‐sup‐0001‐SuppMat.pdf.

**Supporting File 2**: advs74180‐sup‐0002‐FigureS1.jpg.

**Supporting File 3**: advs74180‐sup‐0003‐FigureS2.jpg.

**Supporting File 4**: advs74180‐sup‐0004‐FigureS3.jpg.

**Supporting File 5**: advs74180‐sup‐0005‐FigureS4.jpg.

**Supporting File 6**: advs74180‐sup‐0006‐FigureS5.jpg.

**Supporting File 7**: advs74180‐sup‐0007‐FigureS6.jpg.

**Supporting File 8**: advs74180‐sup‐0008‐FigureS7.jpg.

**Supporting File 9**: advs74180‐sup‐0009‐FigureS8.jpg.

**Supporting File 10**: advs74180‐sup‐0010‐FigureS9.jpg.

## Data Availability

The data that support the findings of this study are available from the corresponding author upon reasonable request.
