# Supplementary material for: On the Ordering Mechanism of Cu+ in 2D van der Waals Multiferroic CuCrP2S6
Source: Adv Sci (Weinh). 2026 Feb 4;13(21):e24227. doi: 10.1002/advs.202524227 (PMC13073258; doi:10.1002/advs.202524227)
Supplement: Supplementary file 1 — Supporting File 1: advs74180‐sup‐0001‐SuppMat.pdf. [file ADVS-13-e24227-s006.pdf]

Supporting Information: On the ordering mechanism of Cu^{+} in 2D van der Waals multiferroic $CuCrP_2S_6$

Jiasen Guo,[1] Yongqiang Cheng,[1] Michael A. Susner,[2] Ryan P. Siebenaller,[3] Zachary Morgan,[1,*] and Feng Ye[1,†]

[1]*Neutron Scattering Division, Oak Ridge National Laboratory, Oak Ridge, TN 37831, USA*
[2]*Materials and Manufacturing Directorate, Air Force Research Laboratory, Wright-Patterson Air Force Base, OH 45433, USA*
[3]*Department of Materials Science and Engineering, The Ohio State University, Columbus, OH 43210, USA*

1. (3+1)-DIMENSIONAL SUPERSPACE REFINEMENT

Incommensurate structure lacks transitional symmetry in one or more directions in 3D space. For an one-dimensional modulation, the missing symmetry can be recovered by introducing a fourth dimension $\mathbf{x_4}$, orthogonal to the original three directions $(\mathbf{d_i}, i = 1, 2, 3)$. The other three axes in the (3+1)-dimensional superspace, $\mathbf{x_1}$, $\mathbf{x_2}$, $\mathbf{x_3}$ are defined through $\tan(\mathbf{d_i}, \mathbf{x_i}) = \sigma_i|\mathbf{x_4}|/|\mathbf{d_i}|$, with σ_i the component of modulation wavevector $\mathbf{q_s}$ indexed in the 3D reciprocal space [1, 2]. The position of an atom in the superspace is given by

$$\begin{aligned} \bar{\mathbf{r}} &= \mathbf{L} + \mathbf{r_0}, \\ \bar{x}_4 &= t + \mathbf{q_s} \cdot \bar{\mathbf{r}}, \\ \mathbf{r} &= \bar{\mathbf{r}} + \mathbf{u}(\bar{x}_4), \\ x_4 &= \bar{x}_4 + \mathbf{q_s} \cdot \mathbf{u}(\bar{x}_4), \\ x_i &= r_i. \quad (i = 1, 2, 3) \end{aligned} \tag{S1}$$

Here, $\bar{\mathbf{r}}$ is the average position of the atom in the 3D space translated from the position in the first unit cell $\mathbf{r_0}$ by lattice vector $\mathbf{L}$. The actual position $\mathbf{r}$ depends on the modulation function $\mathbf{u}(\bar{x}_4)$, with $\bar{x}_4$ determined through $\mathbf{q_s}$ and the modulation phase t [2]. The modulation phase t is defined to run parallel to $\mathbf{x_4}$ and ranges from 0 (origin of the unit cell) to 1 (origin of the next unit cell). Atoms in the superspace are presented as electron density contours called atomic modulation functions or atomic domains in the $\mathbf{x_4}$-$\mathbf{x_i}$ section. Due to the dependence of $\mathbf{u}(\bar{x}_4)$ on $\mathbf{L}$, crystal structures in neighboring unit cells are correlated through the $\mathbf{q_s}$. In practice, the structure in different unit cells can be accessed via varying t, which effectively collapse the varying structures across neighboring unit cells into frames in a single unit cell. Thus, it is convenient to present the structural modulation as a function of t.

The superspace refinement of the X-ray data at $160 \leq T \leq 185$ K was carried out using JANA2020 [3]. The average structure was first refined by fitting to only the Bragg peaks, followed by fitting to both Bragg and satellite peaks together for the modulated structure. Since only first order satellite peaks were observed, each displacement and occupational degrees of freedom are represented by one harmonic function. No modulation was applied to the atomic displacement parameters (ADP) as it tends to produce negative parameters for certain atoms at particular modulation phases. Complementary harmonic functions are used for Cu^{+} occupancies to ensure the overall unity occupancy within a given CuS_6. For $140 \leq T \leq 155$ K, refinements were only performed for the average structure with the ADP of Cu^{+} sites constrained to be identical in the same octahedron. This avoid negative ADP at the lower occupancy site. Refinement residuals are summarized in Table S1.

Fig. S1 presents the refined displacive modulations of independent atoms at T = 185 K, illustrated in the inset. Occupational modulation of Cu^{+} is evidenced by the varying electron density along the $\mathbf{x_4}$ axis and displacive modulation is only seen along the $\mathbf{x_3}$ axis (Fig. 3a), reflecting nearly vertical displacement along the layer normal. Strong displacive modulations are observed for the S atoms, giving rise to the *breathing* of the S_3 triangles near Cu^{+}. No modulation is observed for the Cr^{3+}. The displacive modulations of the P atoms are expected as the tilting of P_2S_6 is necessary to accommodate the *breathing* of S_3 triangles.

Fig. S2 displays the temperature evolution of lattice parameters. Lattice constants a and b increase continuously in the quasi-antipolar state and change slopes below $T^{\star}$, marking distinct thermal expansion properties of the incommensurate (ICM) phase and the antiferroelectric (AFE) phase. In contrast, lattice constant c linearly increases through the whole temperature range independent on Cu^{+} ordering, showing an anisotropic thermal expansion. The monoclinic angle β appears to be less sensitive to the onset of AFE phase, which increases gradually from T_{c1} with a linear trend through $T^{\star}$. At T_{c2}, β reaches its maximum followed by a slight decrease in the AFE state. These observations highlight a negative thermal expansion effect, which drives the weak expansion of the S_3 triangles and Cu^{+} shift in the Γ_1^{+} mode along the layer normal given in Figs. 3e-f.

TABLE S1. Summary of the superspace refinement

T (K)	140	145	150	155	160	165	170	175	180	185
R_{main} (%)	3.67	3.77	4.29	4.96	3.76	3.71	3.77	4.12	4.61	5.12
$R_{satellite}$ (%)	N/A	N/A	N/A	N/A	9.24	8.69	8.08	8.76	9.06	11.13
number of refined parameters	200	200	200	200	86	86	86	86	86	86
number of reflections	6229	6228	6229	6220	4766	4766	4769	4616	4772	4769
space group	P_c				$C2/c(\alpha, 0, \gamma)00$					

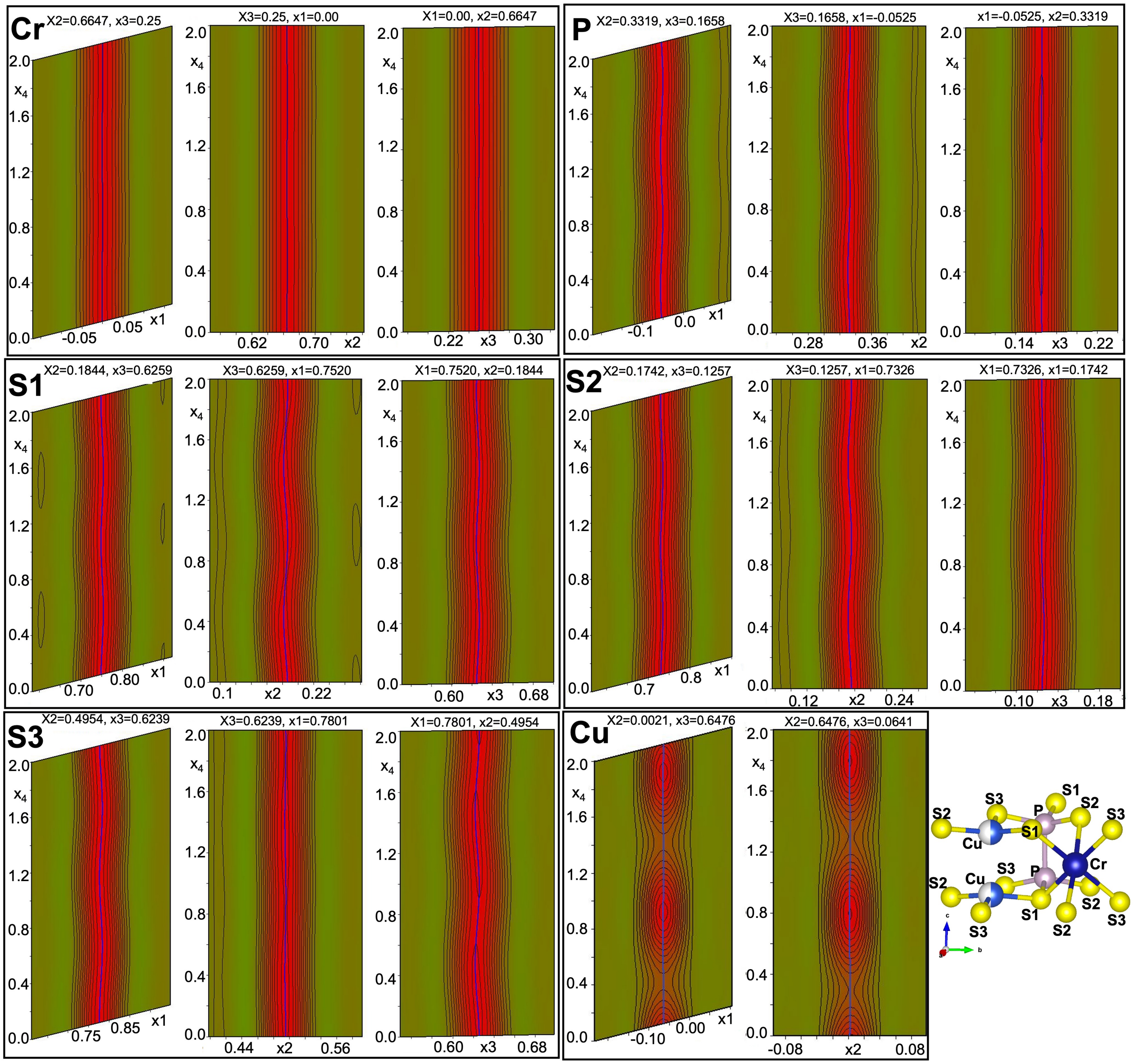

FIG. S1. Superspace electron density sections of crystallographically-inequivalent atoms. Red and green color represent positive and negative electron density and solid black lines are isocountours. Solid blue lines are the refined atomic modulation functions. The inset illustrates CuS_6, CrS_6 and P_2S_6 for atom label references.

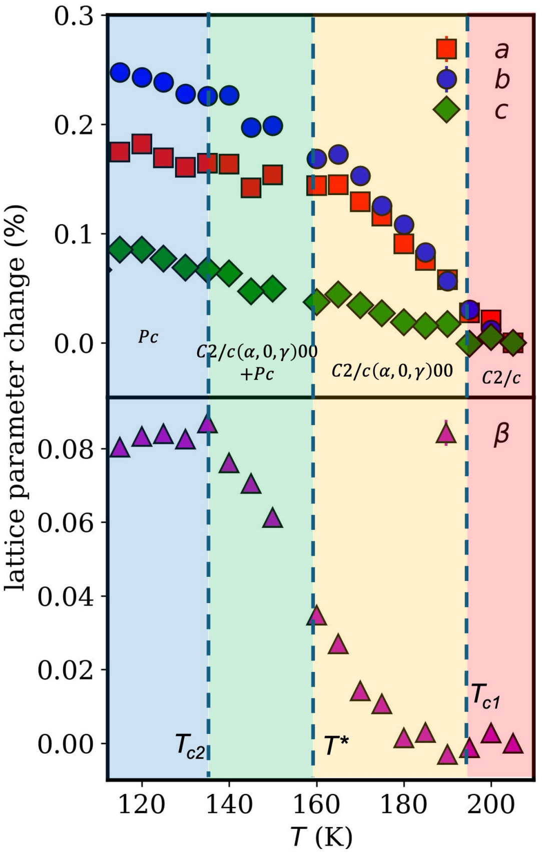

FIG. S2. Temperature evolution of the lattice constants a, b, c and the unique β angle.

2. DISTORTION SUBGROUP ANALYSIS

The distortion subgroup analysis is performed using the ISODISTORT software tool [4, 5]. The high temperature paraelectric phase ($C2/c$) is taken as the parent structure and the ICM phase ($C2/c(\alpha, 0, \gamma)00$) is treated as the child structure. The Irreducible Representation (IR) B_1 is selected with $\mathbf{q_s} = (\alpha, 0, \gamma)$, resulting in two distortion modes $B_1(\alpha, 0, \gamma)$ and $\Gamma_1^+(0, 0, 0)$.

With one harmonic function describing the displacive modulation of each Cartesian degree of freedom, the refined modulated structure contains 49 displacive parameters, corresponding to 49 local displacive modes, including 16 Γ_1^+ modes and 33 B_1 modes. Conversion from the refined structure parameters to the local mode amplitudes is achieved via

$$\Delta = \mathbf{M}(\mathbf{AN}), \quad \mathbf{A} = \left((\mathbf{M}^{-1})\Delta\right)/\mathbf{N}, \tag{S2}$$

where Δ and $\mathbf{A}$ denote column vectors of refined structure parameters and local mode amplitudes, respectively. Here, $\mathbf{M}$ is the temperature-dependent 49×49 transformation matrix and $\mathbf{N}$ is the column vector of normalization factors, both generated by ISODISTORT. Similarly, the occupational local mode amplitudes can be reconstructed. Figs. S3 and S4 show the temperature evolution of the individual local mode amplitude. The amplitudes presented in Figs. 3g-i of the main text are defined as $\sqrt{\sum_i a_i^2}$ with a_i being the local distortion mode amplitude of each inequivalent site of the same atom type.

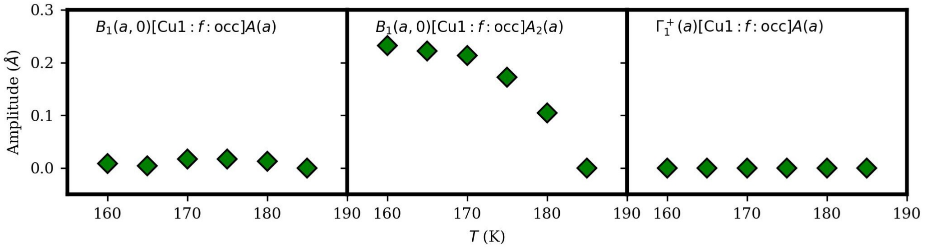

FIG. S3. Temperature evolution of the local occupational mode amplitudes in Γ_1^+ and B_1 distortion modes, relative to $T = 185$ K. The local mode are named following the convention in Reference [4]. Only the B_1 mode contributes to the Cu^+ occupational change.

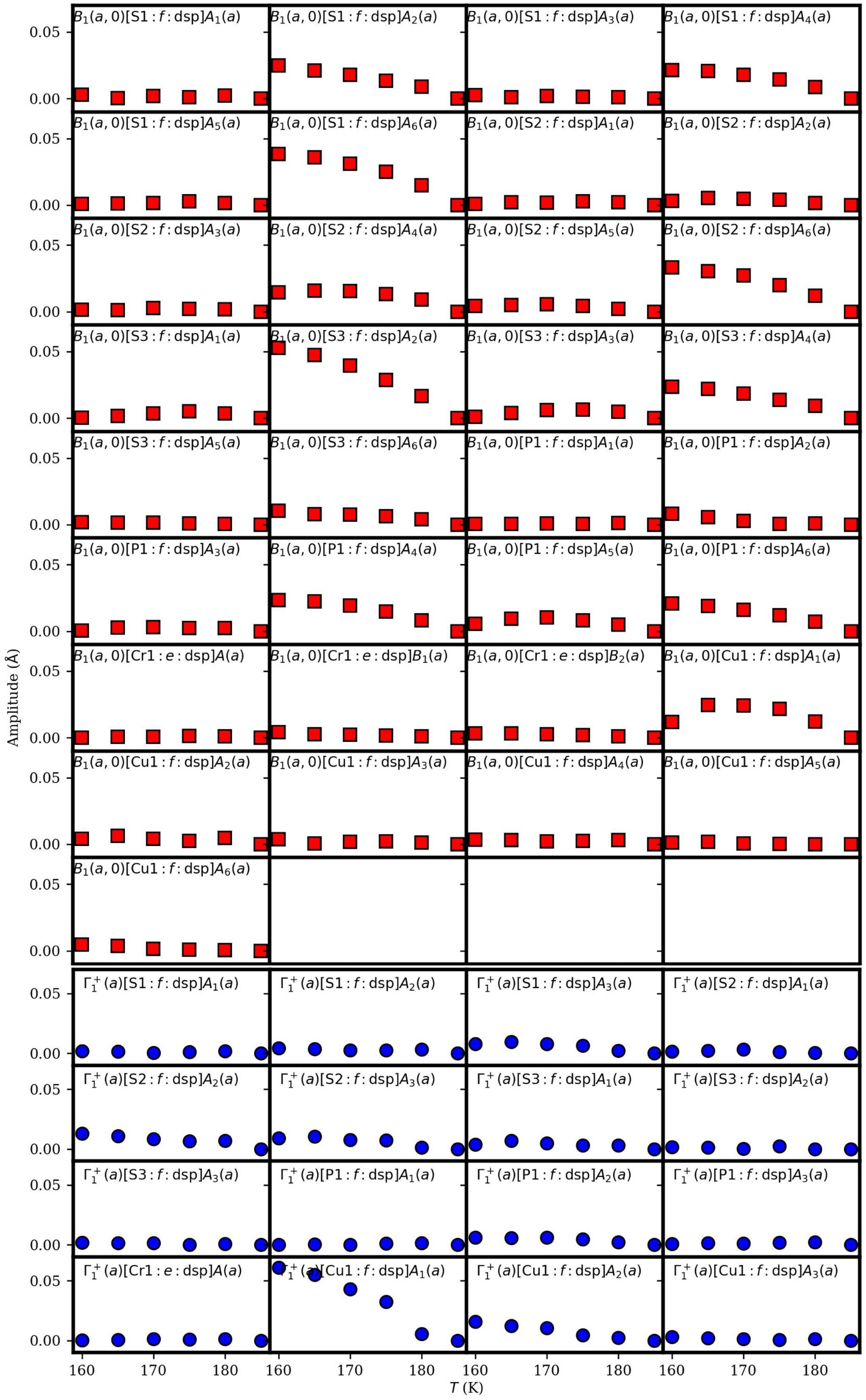

FIG. S4. Temperature evolution of the local displacive mode amplitudes in Γ_1^+ and B_1 distortion modes, relative to $T = 185$ K. The local mode are named following the convention in Reference [4]. Cu^+ displacements are mostly found in the Γ_1^+ mode, S displacements are mostly contributed by the B_1 mode.

3. 3D-ΔPDF ANALYSIS

Starting from the X-ray diffuse scattering data symmetrized for enhancing statistics, the three-dimensional difference pair distribution function (3D-ΔPDF) analysis was carried out by first removing integer Bragg peaks and scattering artifacts through a k-space algorithm involving a moving window [6]. For each window, the voxel at the window center was labeled as an outlier if the voxels value is larger than a threshold times the median of the remaining data in the window [7]. A punch-and-fill process was followed to label the remnant integer Bragg intensities as outliers. The outlier voxels were then back-filled with a Gaussian filter ($\sigma = 5$) interpolation. 3D-ΔPDF was calculated with the diffuse scattering signal using *NumPy* discrete Fourier transform library, followed by a convolution with a Gaussian filter ($\sigma = 2.5$) to remove the Fourier ripples.

Figs. S5a-c present 3D-ΔPDF patterns at T = 206 K. Positive and negative correlations are colored into red and blue. Labeled arrows map PDF peaks to specific Cu^+-Cu^+ bonds in CCPS, demonstrated in Figs. S5d-f. The temperature evolution of the structural modulation in the quasi-antipolar state is also revealed in the 3D-ΔPDF analysis, shown in Fig. S6.

Figs. S7 shows the estimation of Cu^+ correlation length along the b direction at T = 230, 215 and 195 K from real space 3D-ΔPDF analysis and reciprocal space analysis. The real space correlation length was obtained by fitting the positive peaks in the y-direction line cut profile using

$$I \propto \exp\left(-|y|/\xi\right) \times \exp\left(-y^2/2\sigma^2\right) + BG, \tag{S3}$$

where ξ is the correlation length and BG stands for background. σ is the Gaussian envelope width evaluated from fitting to 3D-PDF peak intensity decay at T = 100 K [8]. The reciprocal space analysis was carried out by fitting K-direction line cut profile across the diffuse signal with a Lorentzian form

$$I \propto 1/(q^2 + \kappa^2) + BG, \tag{S4}$$

where κ is the inverse correlation length 1/ξ. The estimated correlation length are labeled in the figure and good agreements are obtained. Fig. S8 compares the extracted correlation lengths along the b and c directions (from reciprocal space), which exhibit similar trends across the transition temperature T_{c1} = 195 K. This indicates that the ordering of Cu^+ is three-dimensional and the vdW gap does not play a role in this process.

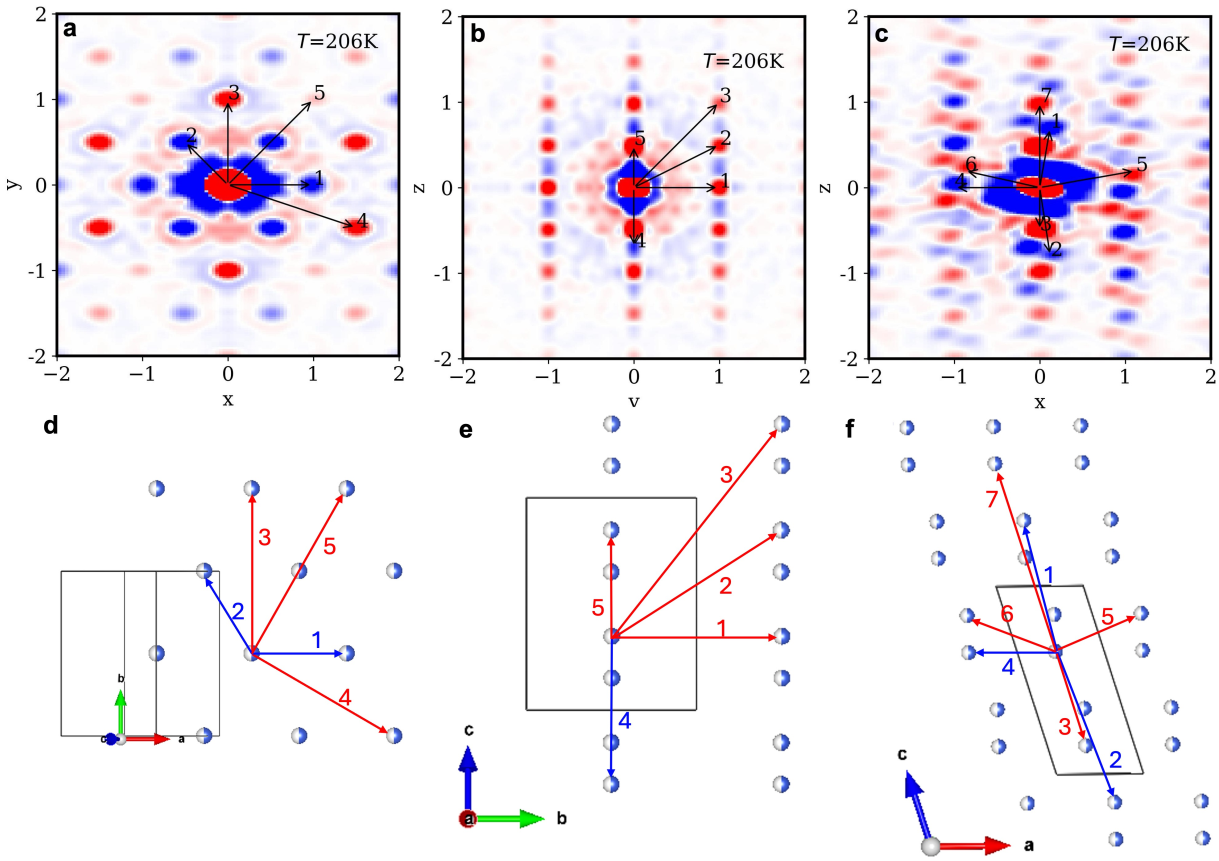

FIG. S5. 3D-ΔPDF analysis of the diffuse scattering signal at T = 206 K and peak-bond mapping. 3D-ΔPDF patterns in the (a) ab, (b) bc and (c) ac planes, respectively, in fractional coordinates. For the bc plane, $-0.02 < x < 0.14$ are integrated to include the inter- and intra-CuS_6 correlations in the same plot. Positive and negative correlations are colored into red and blue. Peak-bond mappings in the (d) ab, (e) bc and (f) ac planes. For clarity, only Cu^+ sites are shown. Colored arrows and labels mark the positive (red) and negative (blue) correlations between sites at this temperature.

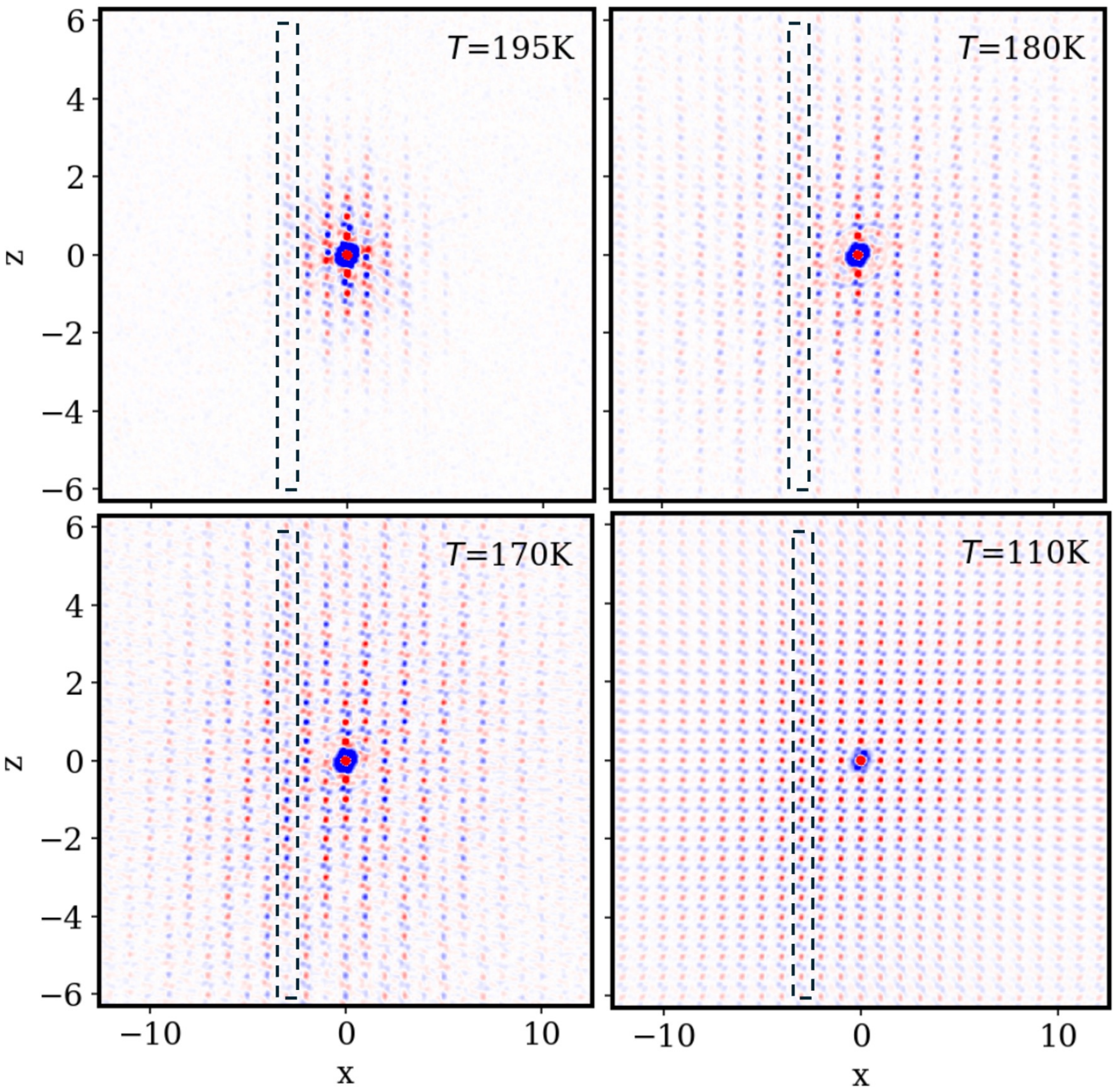

FIG. S6. Temperature evolution of structural modulation in the a and c directions revealed by 3D-ΔPDF analysis. As the temperature decreases, the ICM structural modulation becomes clearly visible with correlation peaks changing from positive to negative along the z direction, highlighted in the dashed rectangles. The modulation becomes increasingly prominent as the temperature decreases. At T = 110 K, the long-range commensurate order establishes.

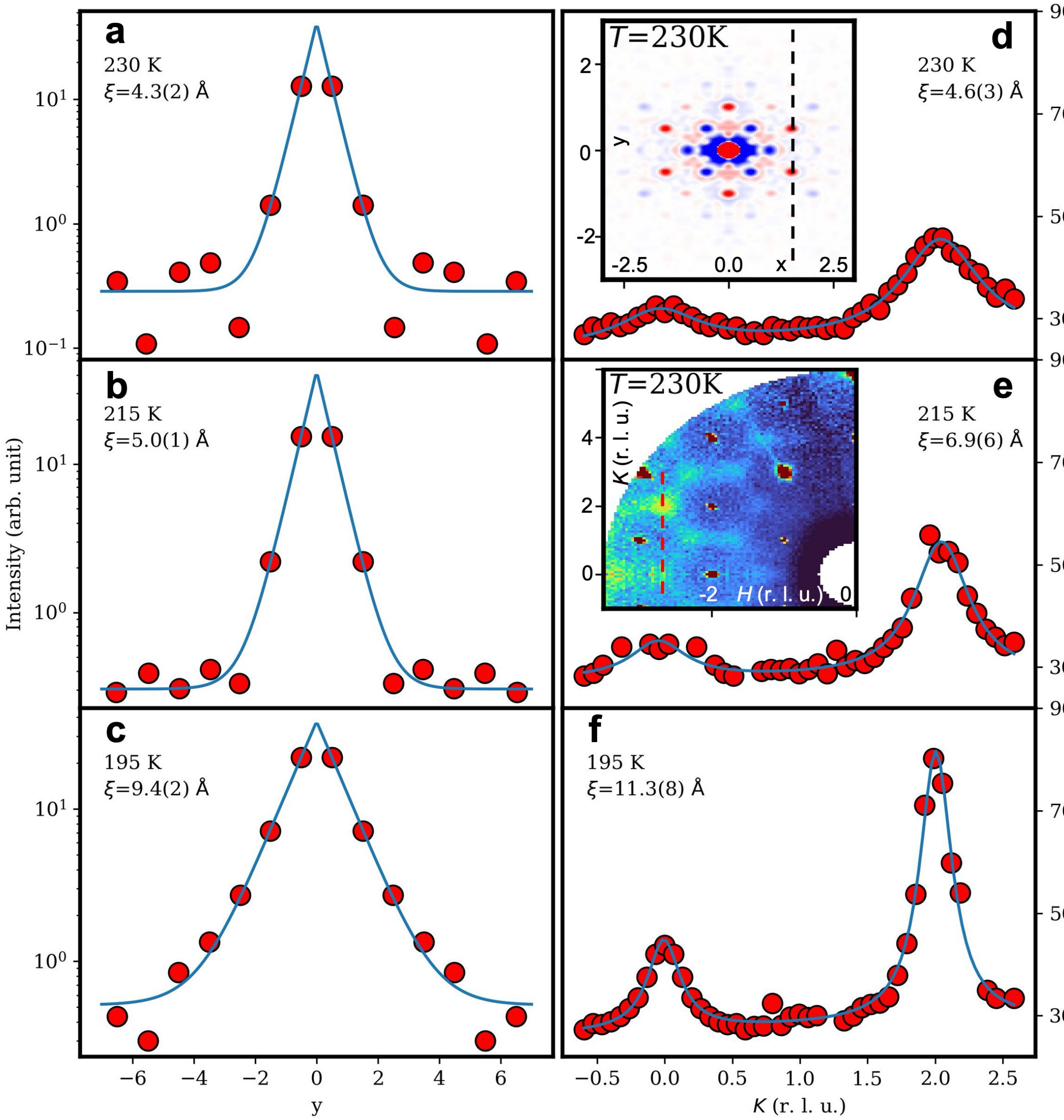

FIG. S7. Estimation of the correlation length among Cu^{+}. Line cut profiles along the b direction on the ab plane of the real space 3D-ΔPDF map at (a) T = 230 K, (b) T = 215 K and (c) T = 195 K. Inset in (d) shows the line cut. The solid line is the model fit given by Equation S3. (d) to (f) Line cut profiles along the K direction in the reciprocal space at mentioned temperatures, illustrated in the inset in (e). The solid line is a Lorentzian line shape fit.

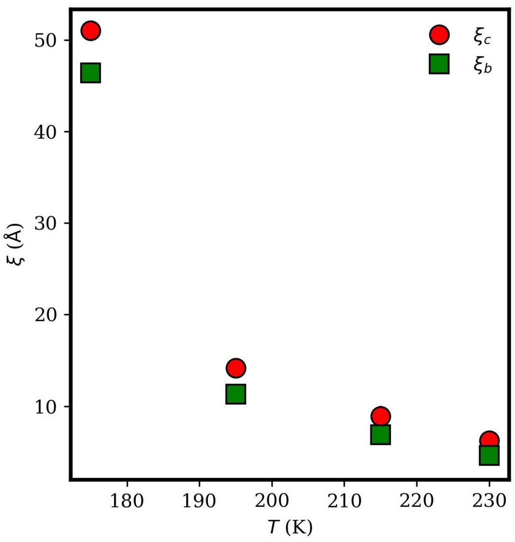

FIG. S8. A comparison between the temperature evolution of the correlation length along b and c.

4. RAMAN SPECTRA CALCULATED BY DENSITY FUNCTIONAL THEORY

Fig. S9 presents the Density Functional Theory (DFT) calculations simulated Raman spectra overlaid with the experimental spectra. Good matches between the observed and predicted peak frequencies were obtained for the characteristic peaks at ∼ 200, 266, 381, and 600 cm^{-1}. These peaks corresponds to the rotations (R'), translations (T'), symmetric stretching (ν_1) and deformation (ν_2), respectively, of the PS_3 group in the P_2S_6 octahedron [9], illustrated in the insets. Importantly, a low frequency mode at ∼ 48 cm^{-1} was also predicted. This mode features displacement of the Cu^+ along the layer normal and the *breathing* motion of the nearby S_3. Based on the similarity between the atomic displacement pattern of this Raman mode and that of the B_1 distortion mode from isodistort analysis (Fig. 3j), we suspect this Raman mode evolves from the amplitudon of the ICM phase. The B_1 distortion mode describes the Cu^+ redistribution and the anti-phase *breathing* of the surrounding S_3 triangles, which can be effectively viewed as the eigenvector of the amplitudon. As the temperature tunes the structural modulation, it also changes the periodicity of the amplitudon, with its eigenvector maintaining the B_1 symmetry. Upon the establishment of the commensurate phase, the amplitudon develops into the Γ point Raman mode under consideration, with the B_1 symmetry-adapted atomic displacement largely inherited.

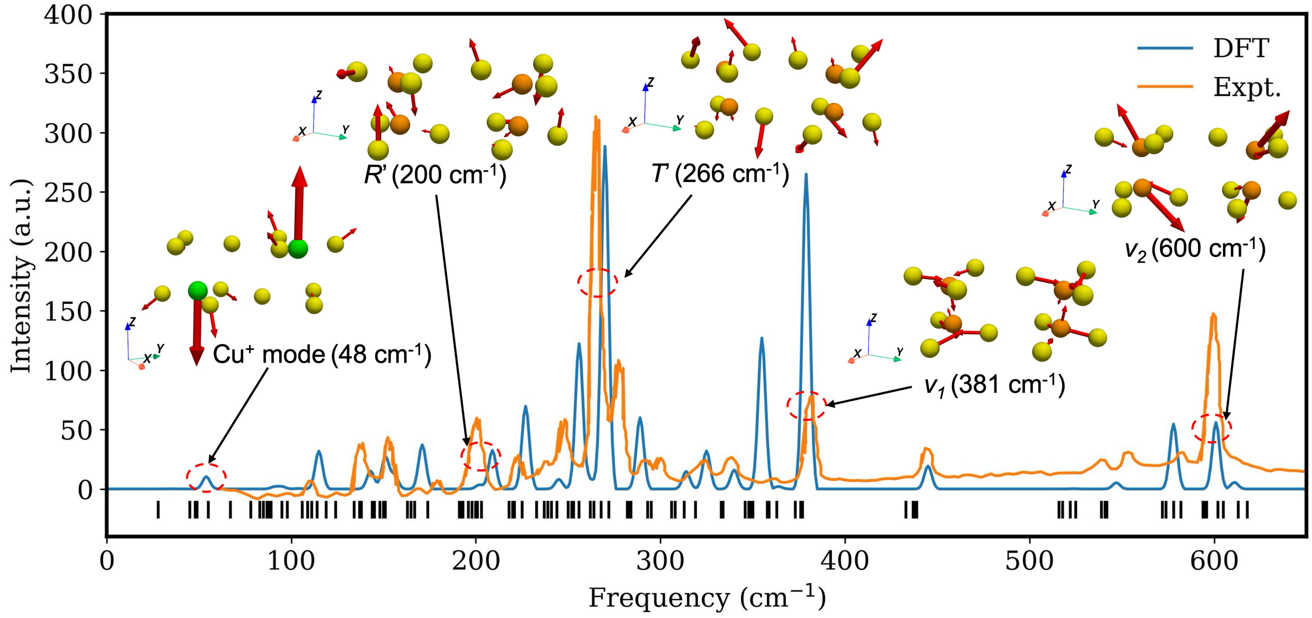

FIG. S9. DFT predicted Raman spectra in the AFE state overlay with the experimental spectra at T = 100 K. Insets illustrate the atomic displacement pattern of characteristic modes. Red arrows denote eigenvectors, which are scaled by a factor of 3 for the S atoms in the Cu^+ mode. In all illustrations, only the relevant atoms are shown (Cu^+ in green, S in yellow, P in orange). The DFT predicted spectra is scaled by a factor of 1.06 and Gaussian broadened to match with the experimental spectra.

* zgf@ornl.gov
† 2fy@ornl.gov

[1] T. Wagner and A. Schönleber, A non-mathematical introduction to the superspace description of modulated structures, Acta Cryst. B **65**, 249 (2009).

[2] S. van Smaalen, An elementary introduction to superspace crystallography, Z. Kristallogr. Cryst. Mater. **219**, 681 (2004).

[3] V. Petříček, L. Palatinus, J. Plášil, and M. Dušek, Jana2020 – a new version of the crystallographic computing system Jana, Z. Kristallogr. Cryst. Mater. **238**, 271 (2023).

[4] B. J. Campbell, H. T. Stokes, D. E. Tanner, and D. M. Hatch, *ISODISPLACE*: a web-based tool for exploring structural distortions, J. Appl. Cryst. **39**, 607 (2006).

[5] H. T. Stokes, D. M. Hatch, and B. J. Campbell, Isodistort, isotropy software suite.

[6] J. Weng, E. D. Dill, J. D. Martin, R. Whitfield, C. Hoffmann, and F. Ye, K-space algorithmic reconstruction (KAREN): a robust statistical methodology to separate Bragg and diffuse scattering, J Appl Crystallogr **53**, 159 (2020).

[7] Z. J. Morgan, H. D. Zhou, B. C. Chakoumakos, and F. Ye, rmc-discord: reverse Monte Carlo refinement of diffuse scattering and correlated disorder from single crystals, J Appl Cryst **54** (2021), Number: 6 Publisher: International Union of Crystallography.

[8] M. J. Krogstad, S. Rosenkranz, J. M. Wozniak, G. Jennings, J. P. C. Ruff, J. T. Vaughey, and R. Osborn, Reciprocal space imaging of ionic correlations in intercalation compounds, Nat. Mater. **19**, 63 (2020).

[9] M. A. Susner, R. Rao, A. T. Pelton, M. V. McLeod, and B. Maruyama, Temperature-dependent raman scattering and x-ray diffraction study of phase transitions in layered multiferroic $CuCrP_2S_6$, Phys. Rev. Mater. **4**, 104003 (2020).
