# Supplementary figures and images for: On the Ordering Mechanism of Cu+ in 2D van der Waals Multiferroic CuCrP2S6
Source: Adv Sci (Weinh). 2026 Feb 4;13(21):e24227. doi: 10.1002/advs.202524227 (PMC13073258; doi:10.1002/advs.202524227)

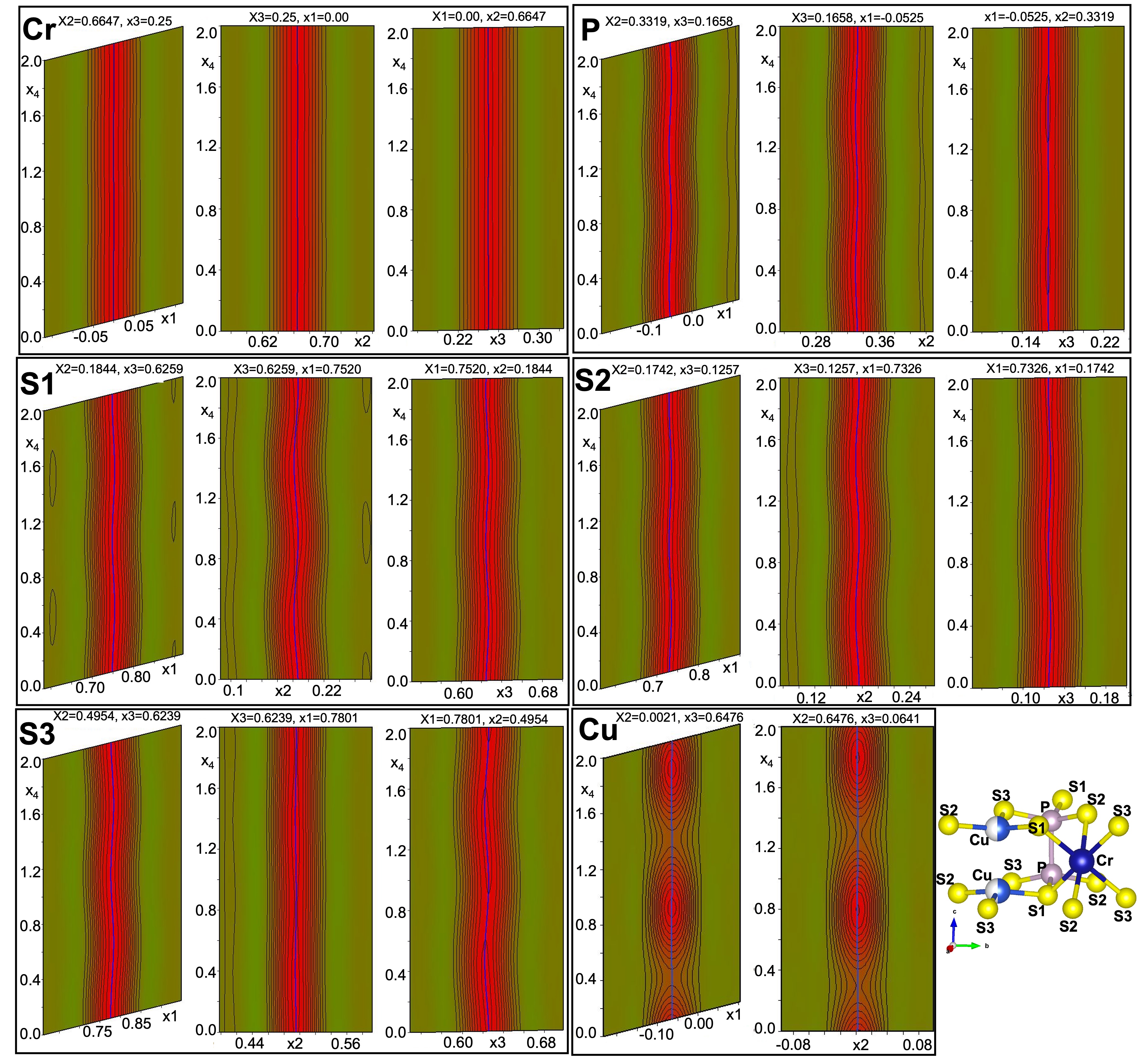

Supplement: Supplementary file 2 — Supporting File 2: advs74180‐sup‐0002‐FigureS1.jpg. [file ADVS-13-e24227-s007.jpg]

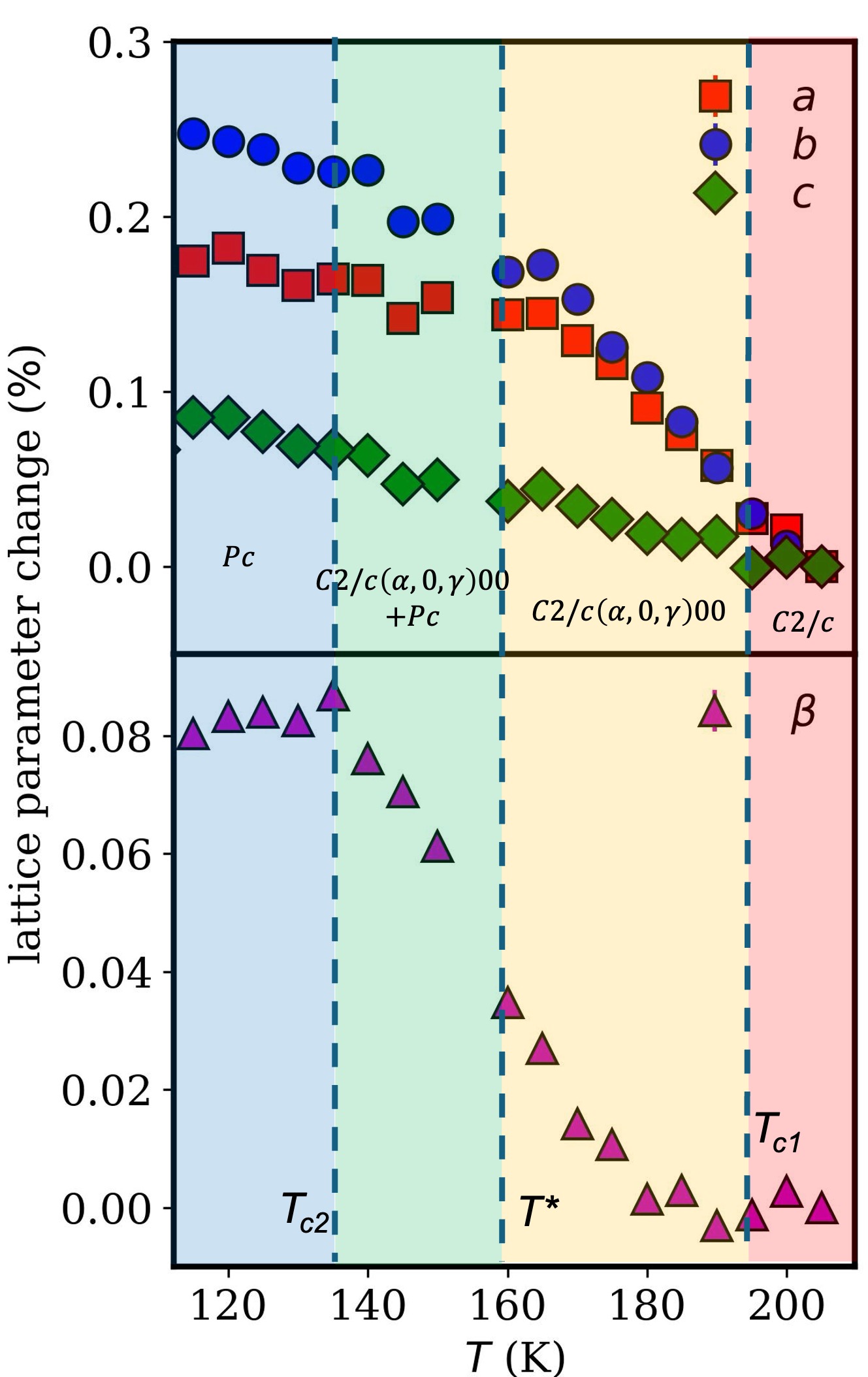

Supplement: Supplementary file 3 — Supporting File 3: advs74180‐sup‐0003‐FigureS2.jpg. [file ADVS-13-e24227-s004.jpg]

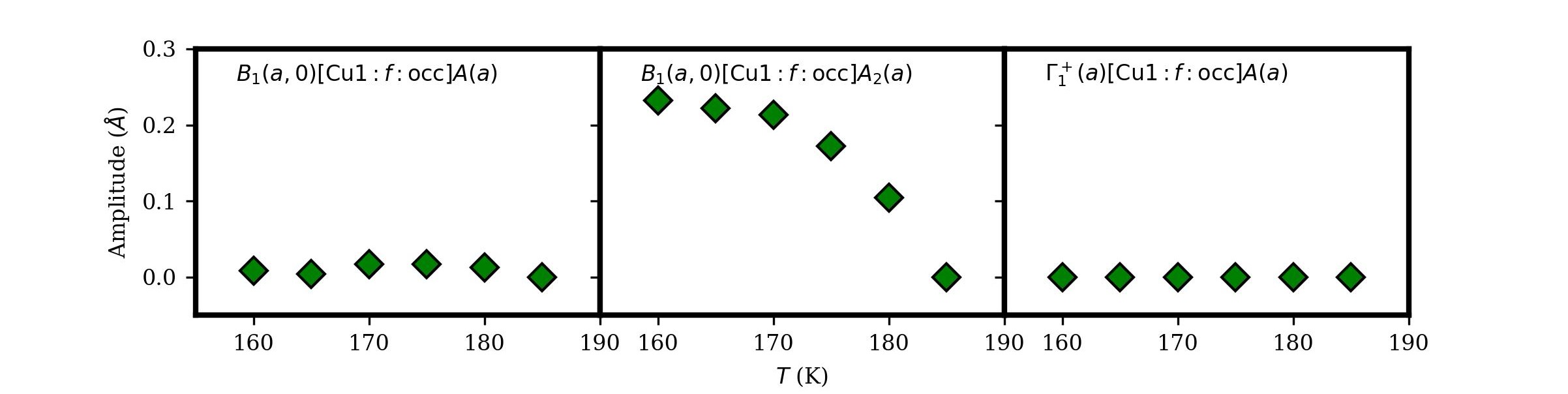

Supplement: Supplementary file 4 — Supporting File 4: advs74180‐sup‐0004‐FigureS3.jpg. [file ADVS-13-e24227-s002.jpg]

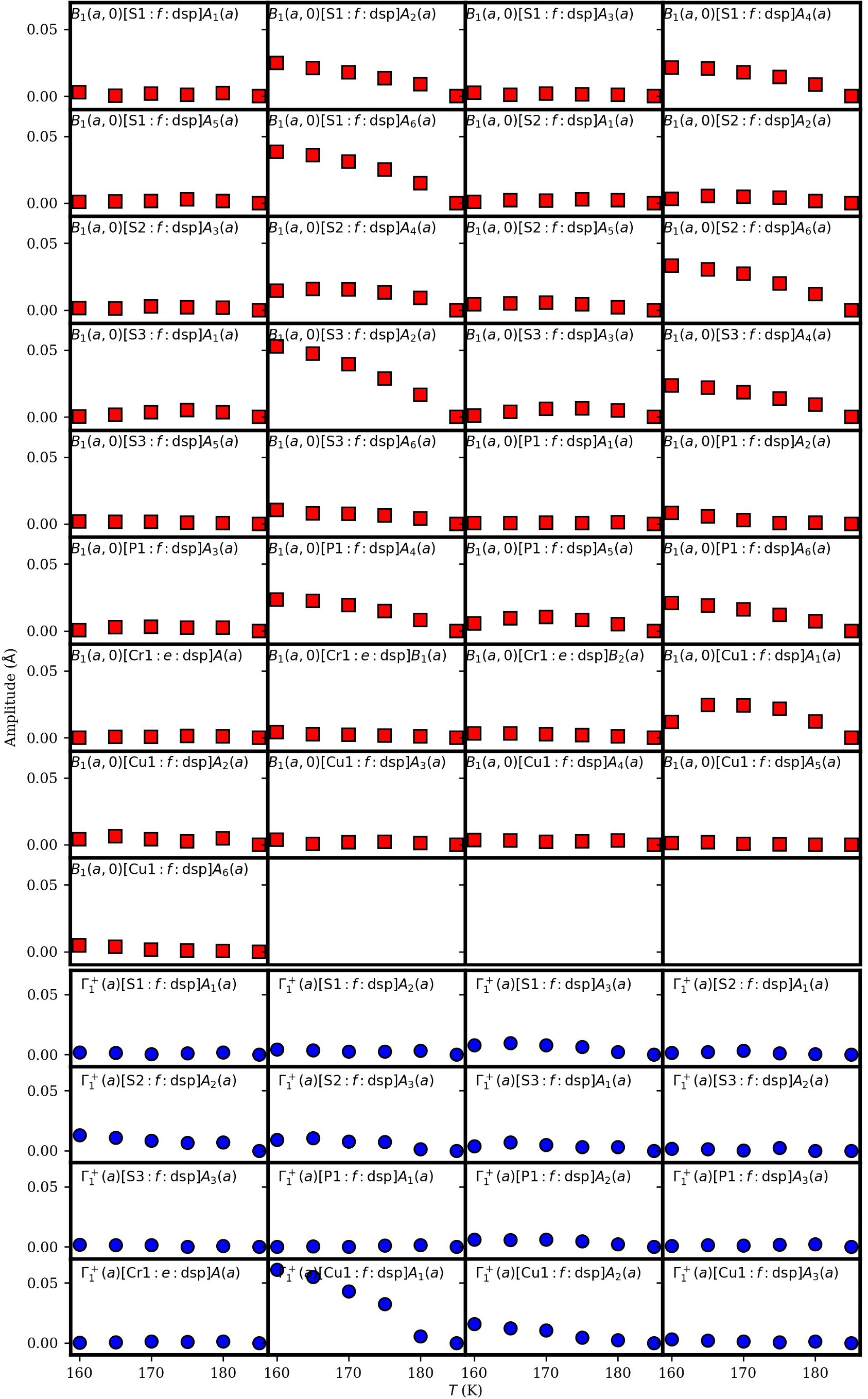

Supplement: Supplementary file 5 — Supporting File 5: advs74180‐sup‐0005‐FigureS4.jpg. [file ADVS-13-e24227-s003.jpg]

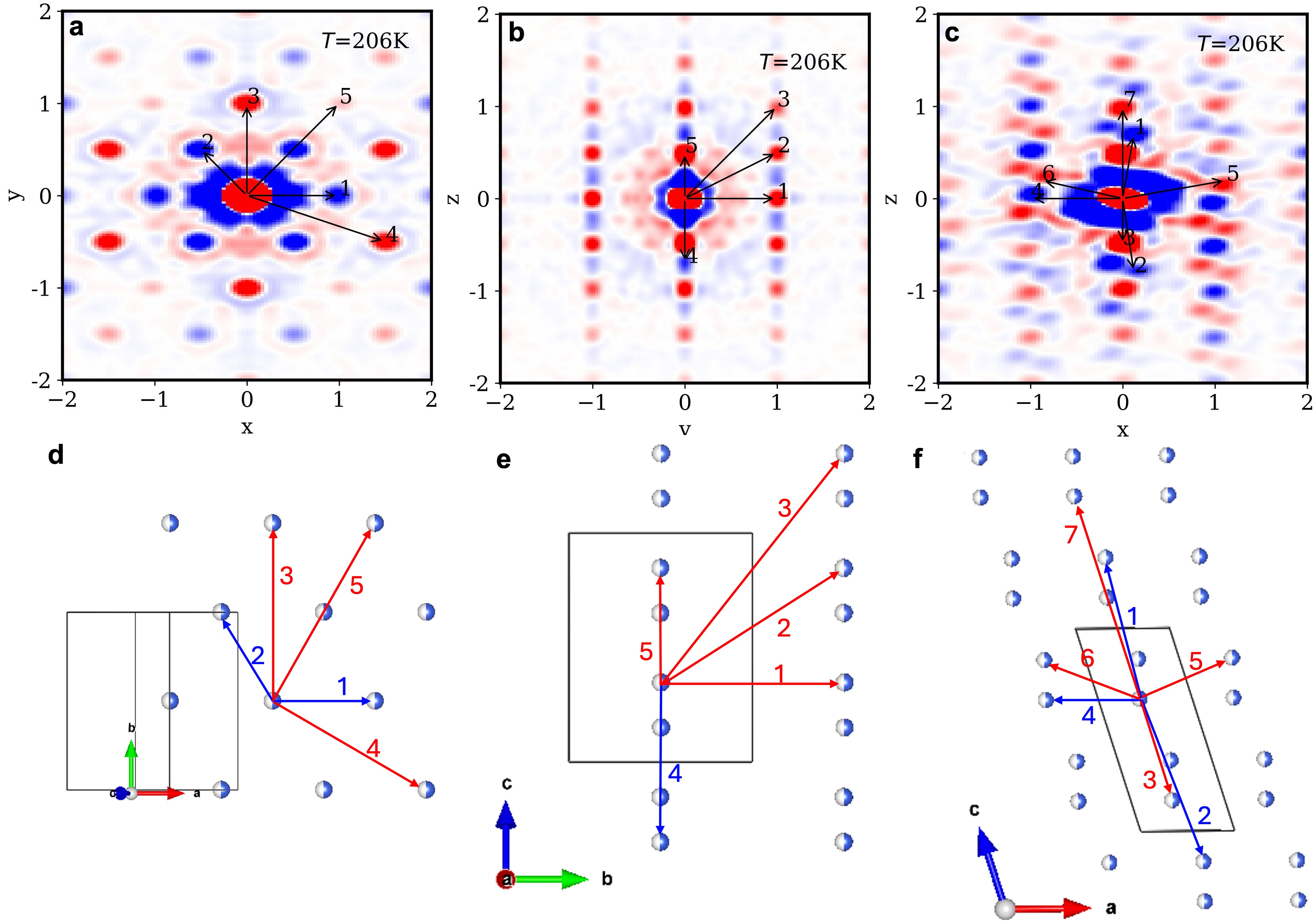

Supplement: Supplementary file 6 — Supporting File 6: advs74180‐sup‐0006‐FigureS5.jpg. [file ADVS-13-e24227-s001.jpg]

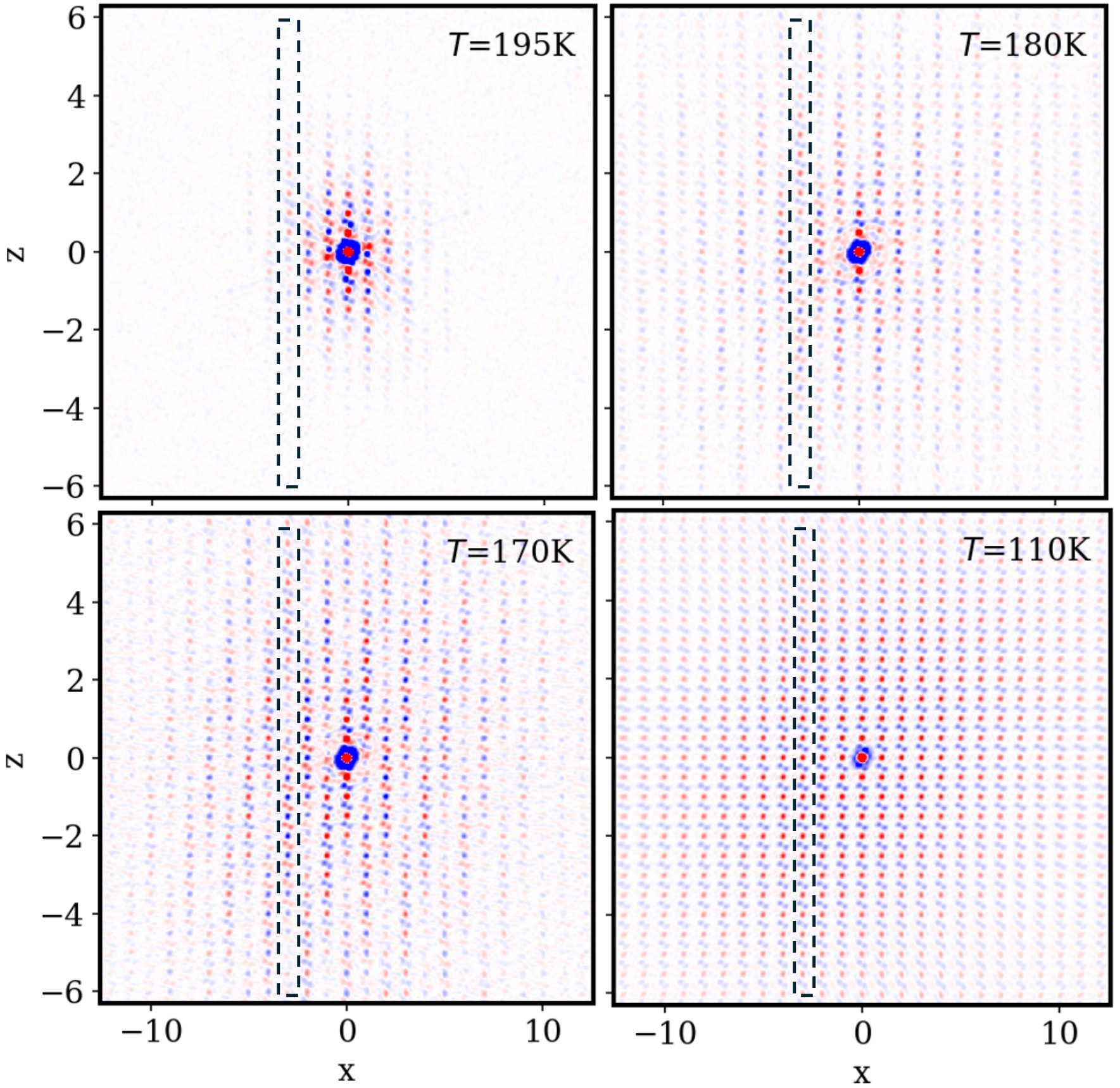

Supplement: Supplementary file 7 — Supporting File 7: advs74180‐sup‐0007‐FigureS6.jpg. [file ADVS-13-e24227-s005.jpg]

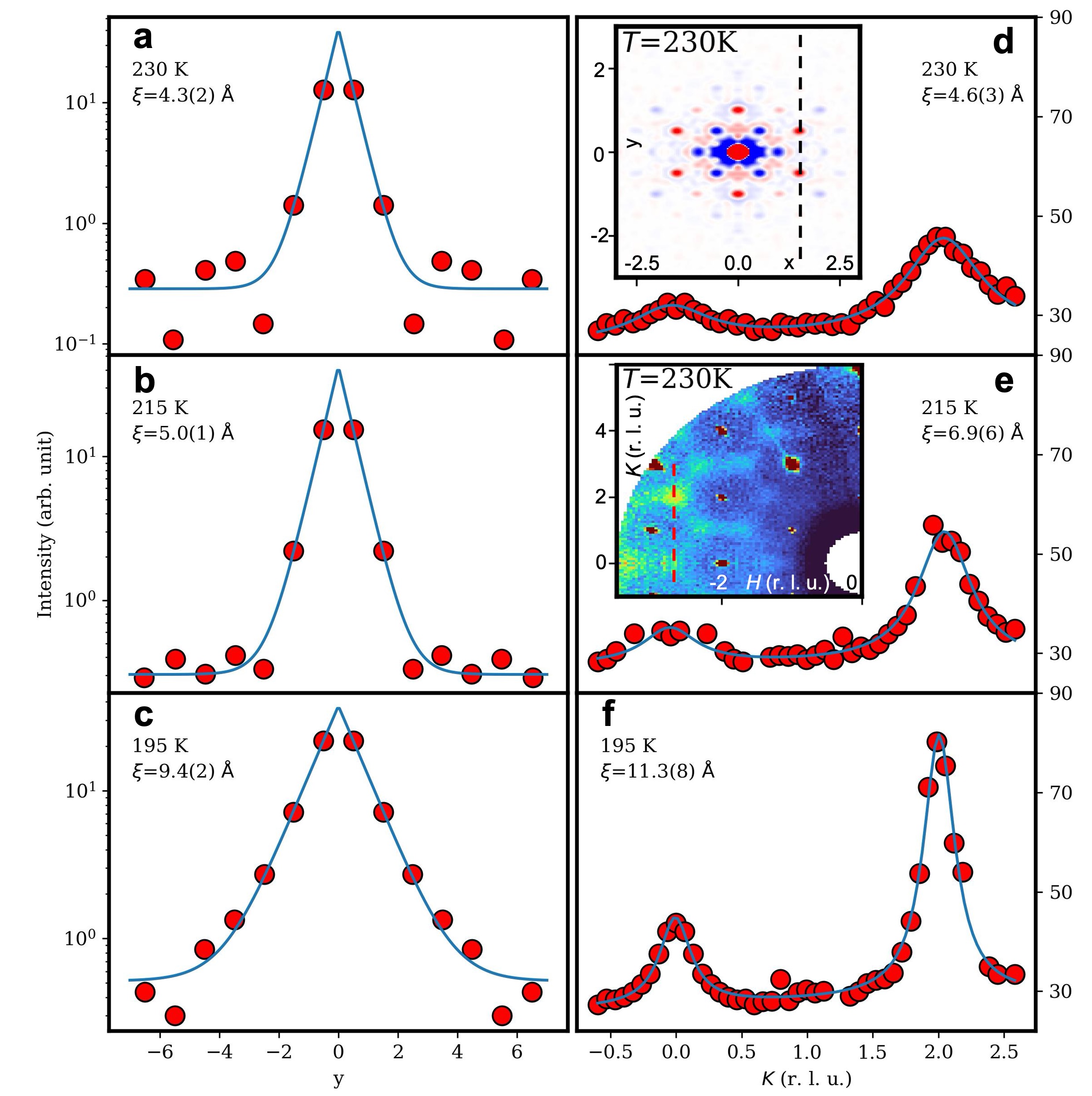

Supplement: Supplementary file 8 — Supporting File 8: advs74180‐sup‐0008‐FigureS7.jpg. [file ADVS-13-e24227-s010.jpg]

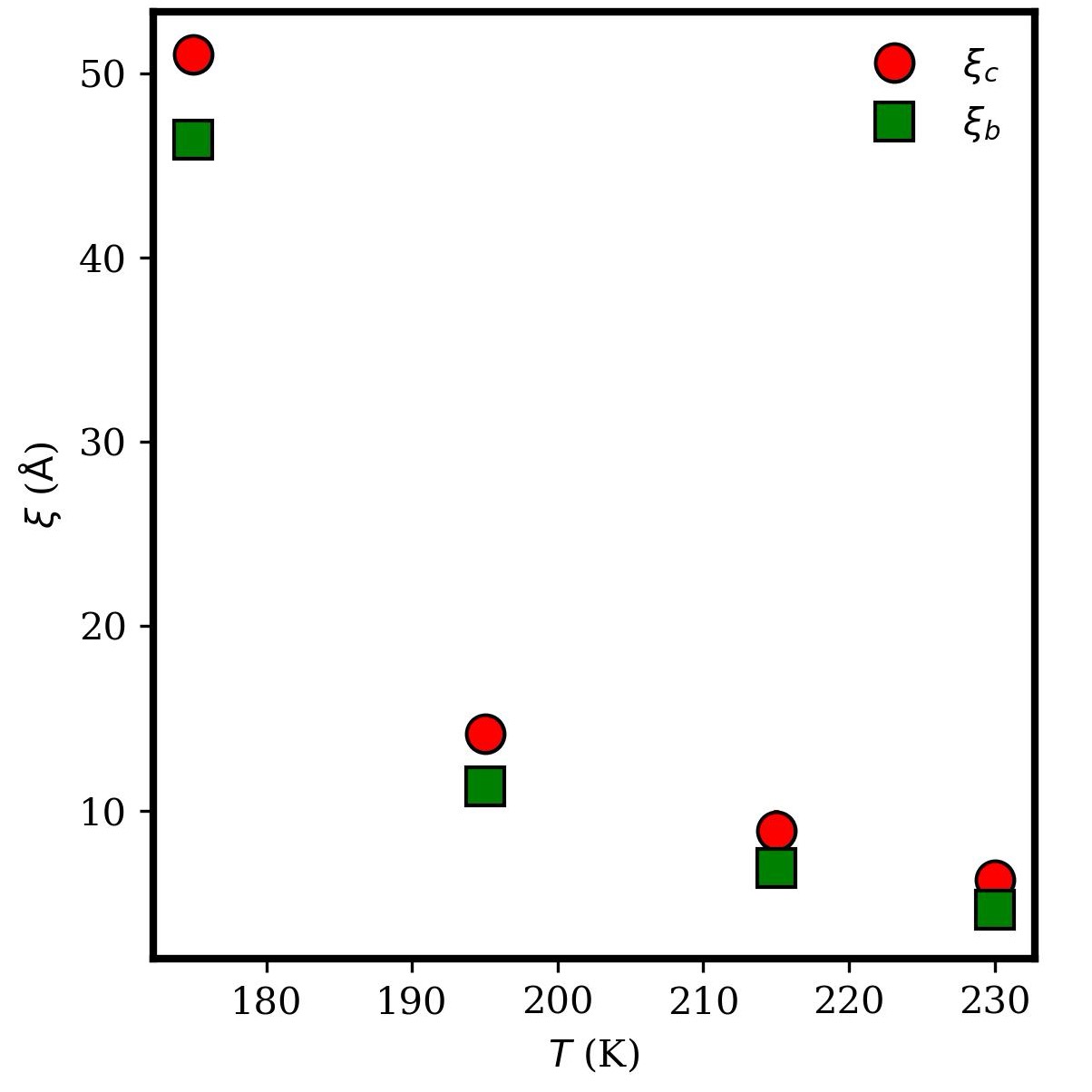

Supplement: Supplementary file 9 — Supporting File 9: advs74180‐sup‐0009‐FigureS8.jpg. [file ADVS-13-e24227-s009.jpg]

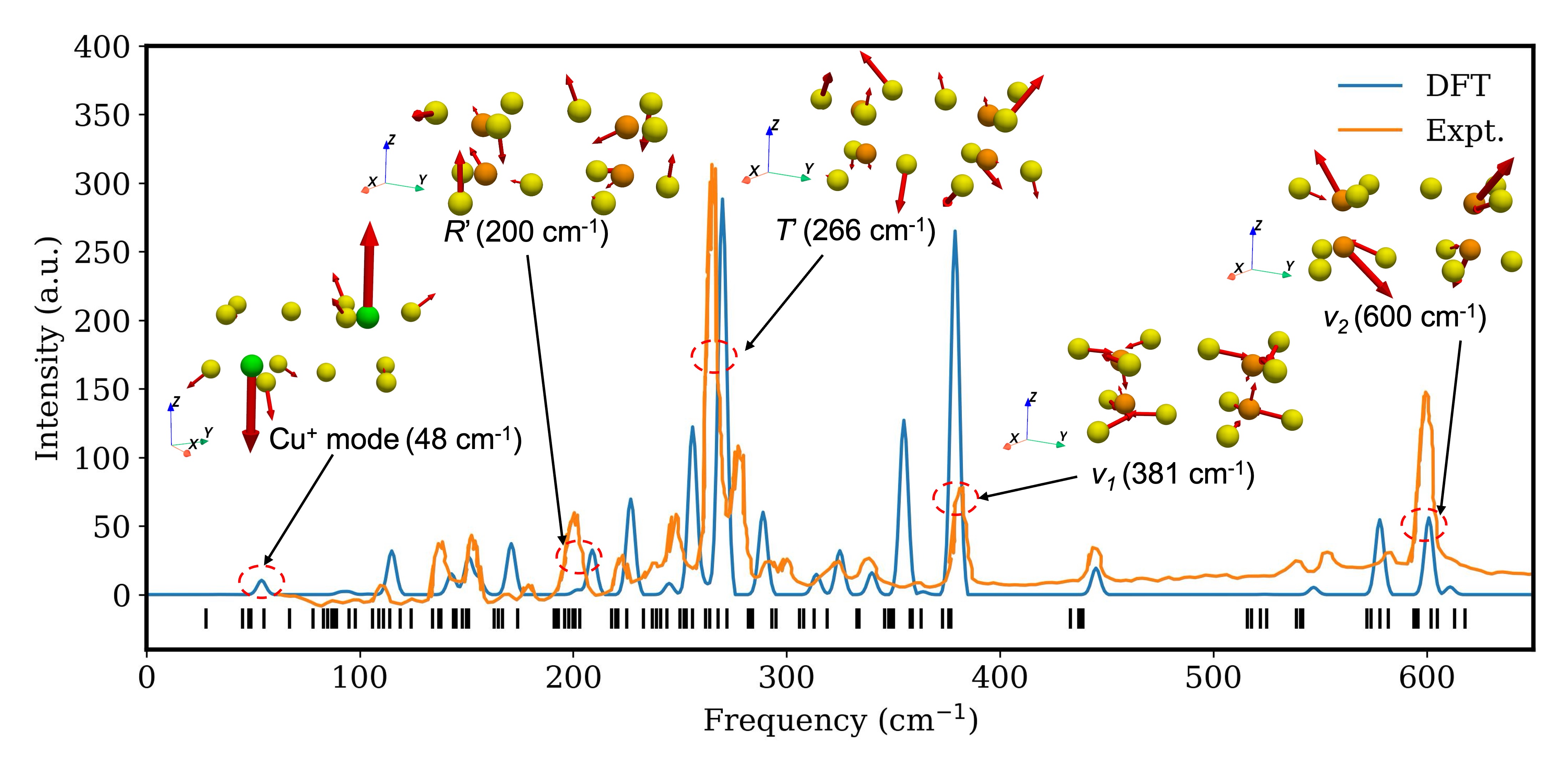

Supplement: Supplementary file 10 — Supporting File 10: advs74180‐sup‐0010‐FigureS9.jpg. [file ADVS-13-e24227-s008.jpg]
